# Multicenter integrated analysis of noncoding CRISPRi screens

**DOI:** 10.1038/s41592-024-02216-7

**Published:** 2024-03-19

**Authors:** David Yao, Josh Tycko, Jin Woo Oh, Lexi R. Bounds, Sager J. Gosai, Lazaros Lataniotis, Ava Mackay-Smith, Benjamin R. Doughty, Idan Gabdank, Henri Schmidt, Tania Guerrero-Altamirano, Keith Siklenka, Katherine Guo, Alexander D. White, Ingrid Youngworth, Kalina Andreeva, Xingjie Ren, Alejandro Barrera, Yunhai Luo, Galip Gürkan Yardımcı, Ryan Tewhey, Anshul Kundaje, William J. Greenleaf, Pardis C. Sabeti, Christina Leslie, Yuri Pritykin, Jill E. Moore, Michael A. Beer, Charles A. Gersbach, Timothy E. Reddy, Yin Shen, Jesse M. Engreitz, Michael C. Bassik, Steven K. Reilly

**Affiliations:** 1 Department of Genetics, Stanford University, Stanford, CA USA; 2 Department of Neurobiology, Harvard Medical School, Boston, MA USA; 3 Departments of Biomedical Engineering and Genetic Medicine, Johns Hopkins University, Baltimore, MD USA; 4 Department of Biomedical Engineering, Duke University, Durham, NC USA; 5 Center for Advanced Genomic Technologies, Duke University, Durham, NC USA; 6 Broad Institute of Harvard & MIT, Cambridge, MA USA; 7 Department of Organismic and Evolutionary Biology, Center for System Biology, Harvard University, Cambridge, MA USA; 8 Harvard Graduate Program in Biological and Biomedical Science, Boston, MA USA; 9 Department of Neurology, Institute for Human Genetics, University of California, San Franscisco, San Francisco, CA USA; 10 University Program in Genetics and Genomics, Duke University School of Medicine, Durham, NC USA; 11 Department of Computer Science, Princeton University, Princeton, NJ USA; 12 Computational and Systems Biology Program, Memorial Sloan Kettering Cancer Center, New York, NY USA; 13 Department of Biology, Duke University, Durham, NC USA; 14 Department of Biostatistics and Bioinformatics, Duke University Medical Center, Durham, NC USA; 15 Department of Electrical Engineering, Stanford University, Stanford, CA USA; 16 Knight Cancer Center, Oregon Health and Science University, Portland, OR USA; 17 The Jackson Laboratory, Bar Harbor, ME USA; 18 Department of Computer Science, Stanford University, Stanford, CA USA; 19 Center for Personal Dynamic Regulomes, Stanford University, Stanford, CA USA; 20 Department of Applied Physics, Stanford University, Stanford, CA USA; 21 Chan Zuckerberg Biohub, San Francisco, CA USA; 22 Howard Hughes Medical Institute, Chevy Chase, MD USA; 23 Department of Immunology and Infectious Disease, Harvard T.H. Chan School of Public Health, Boston, MA USA; 24 Lewis-Sigler Institute for Integrative Genomics, Princeton University, Princeton, NJ USA; 25 Program in Bioinformatics and Integrative Biology, RNA Therapeutics Institute, University of Massachusetts Chan Medical School, Worcester, MA USA; 26 Department of Neurology, University of California, San Francisco, San Francisco, CA USA; 27 Weill Institute for Neurosciences, University of California, San Francisco, San Francisco, CA USA; 28 BASE Initiative, Betty Irene Moore Children’s Heart Center, Lucile Packard Children’s Hospital, Stanford, CA USA; 29 The Novo Nordisk Foundation Center for Genomic Mechanisms of Disease, Broad Institute of MIT and Harvard, Cambridge, MA USA; 30 Department of Genetics, Yale University, New Haven, CT USA

**Keywords:** Gene expression profiling, Genomic analysis

## Abstract

The ENCODE Consortium’s efforts to annotate noncoding *cis*-regulatory elements (CREs) have advanced our understanding of gene regulatory landscapes. Pooled, noncoding CRISPR screens offer a systematic approach to investigate *cis*-regulatory mechanisms. The ENCODE4 Functional Characterization Centers conducted 108 screens in human cell lines, comprising >540,000 perturbations across 24.85 megabases of the genome. Using 332 functionally confirmed CRE–gene links in K562 cells, we established guidelines for screening endogenous noncoding elements with CRISPR interference (CRISPRi), including accurate detection of CREs that exhibit variable, often low, transcriptional effects. Benchmarking five screen analysis tools, we find that CASA produces the most conservative CRE calls and is robust to artifacts of low-specificity single guide RNAs. We uncover a subtle DNA strand bias for CRISPRi in transcribed regions with implications for screen design and analysis. Together, we provide an accessible data resource, predesigned single guide RNAs for targeting 3,275,697 ENCODE SCREEN candidate CREs with CRISPRi and screening guidelines to accelerate functional characterization of the noncoding genome.

## Abstract (web summary)

This analysis provides 108 noncoding CRISPR screens collated by the ENCODE4 consortium and establishes experimental guidelines for future CRISPRi screens characterizing functional *cis*-regulatory elements.

## Main

The noncoding genome contains critical regulators of gene expression and harbors >90% of trait-associated human genetic variation^1–4^. Major efforts over the past decade have mapped hundreds of thousands of noncoding candidate *cis*-regulatory elements (cCREs)^5–7^. Such efforts have relied primarily on mapping sequence conservation and biochemical markers that are correlated with regulatory activity rather than direct functional characterization. Site-specific, programmable and highly scalable CRISPR genome and epigenome manipulation methods have enabled massively parallel perturbation assays to identify and characterize functional CREs. However, the overlap between CREs, elements with empirically characterized endogenous function, and cCREs, elements nominated by biochemical markers, screens or sequence content, is unknown.

Various CRISPR-based perturbation methods have been developed to determine the effects of different cCREs on target gene expression and/or downstream phenotypes^8–14^. Systematic benchmarking of noncoding CRISPR screening methods and attempts to harmonize the results have been limited by low numbers of available datasets and inconsistent reporting. The ENCODE4 Functional Characterization Centers have generated the largest collective dataset of endogenous cCRE perturbation screens to date, including many loci perturbed to saturation in K562 cells, using diverse experimental approaches. Here, we compare noncoding CRISPR screening approaches and provide technical suggestions and data file formats potentially generalizable to such screens. We analyze various CRISPR noncoding screens extensively in K562 cells and other biological systems at each screening stage, including (1) library design, (2) CRISPR perturbation selection, (3) phenotyping strategy and (4) analytical methods. By assembling and jointly analyzing this large repository of bulk CRISPR screens, we develop suggestions for study design, analysis and validation of experiments in these model systems and provide comprehensive benchmarking between methodologies. We demonstrate how experimental parameters can be tuned to address technical limitations. Finally, we leverage our combined analysis of 107 distinct CRISPR screens to interrogate broader properties of gene regulation.

## Results

### The ENCODE noncoding CRISPR database reveals CRE features

**Fig. 1:**
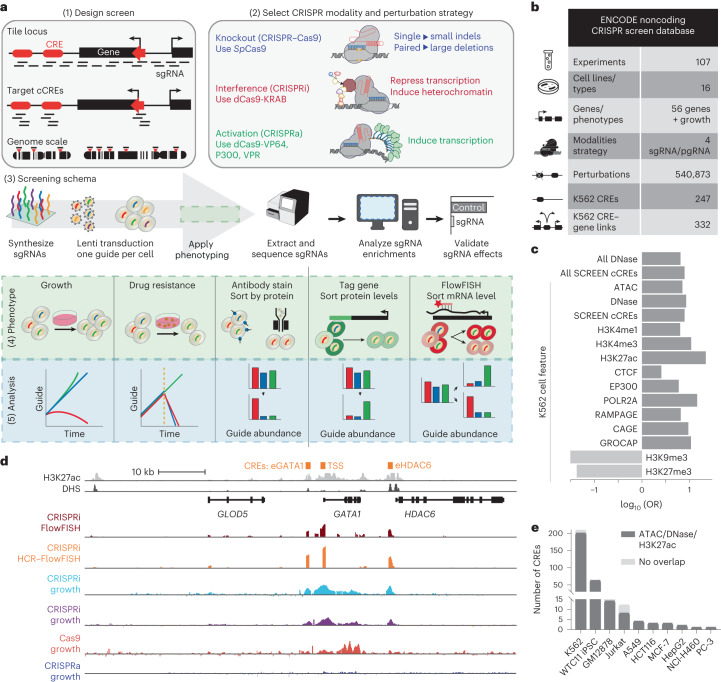
The ENCODE noncoding CRISPR screening database. **a**, CRISPR noncoding strategies including (1) perturbation design strategies, (2) CRISPR modality and perturbation strategies, (3) workflow of a standard screen, (4) phenotyping strategies and (5) analysis approaches; *Sp*Cas9, *Streptococcus pyogenes* Cas9; indels, insertions/deletions. **b**, Summary of the CRISPR screen data performed in human cell lines/types from the April 2022 release of the ENCODE portal. ‘Experiments’, ‘Cell lines/types’, ‘Modalities’, ‘Strategy’, ‘Genes/phenotypes’ and ‘Perturbations’ reflect all human CRISPR screens. ‘K562 CREs’ and ‘K562 CRE–gene links’ reflect results of K562-focused analysis; pgRNA, paired sgRNA. **c**, OR for genomic annotation overlap with CRISPR screen-identified regulatory elements (*n* = 210; Methods). ‘All’ refers to cell-agnostic features. K562 refers to cell-type annotations. All ORs were significant at a *P* value of <0.01, and values were log_10_ transformed for visualization (two-sided Fisher’s exact test). **d**, Genome browser snapshot of the *GATA1* locus including H3K27ac (light gray) and DHS signal (dark gray) in K562 cells. CRISPR screen data (signal log_2_ (FC)) for one replicate each of CRISPRi FlowFISH (dark red), CRISPRi HCR–FlowFISH (orange), Tycko et al.^17^ CRISPRi growth (light blue), Fulco et al.^12^ CRISPRi growth (purple), Cas9 growth (red) and CRISPRa growth (dark blue). Previously validated *GATA1* CREs are labeled on top in orange. **e**, The number of CREs that are significant in a CRISPR screen and overlap accessible chromatin regions, defined by ATAC-seq and DNase-seq and/or H3K27ac ChIP–seq peaks (dark gray) or do not overlap those features in ten cell lines (A549: 4/4; GM12878: 14/14; HCT116: 3/3; HepG2: 2/2; Jurkat: 8/12; K562: 200/210; MCF-7: 3/3; NCI-H460: 1/1; PC-3: 1/1; WTC11: 65/66).

We present a diverse set of >100 noncoding CRISPR screens, all of which are available in the ENCODE portal^15^ (see Supplementary Information Section 2) and 35% of which are first published here (Fig. 1a and Supplementary Tables 1–3). The data used in this study include three targeting approaches: (1) unbiased tiling screens that include single guide RNAs (sgRNAs) targeting cCREs and non-cCRE regions within a specific locus (for example, an entire topologically associated domain (TAD))^9,10,16^, (2) screens that select sgRNAs targeting cCREs in a given locus^12,17,18^ and (3) screens that target cCREs in multiple loci or across the genome^19^. Although tiling screens can identify novel CREs that lack epigenetic marks commonly associated with regulatory activity, cCRE-targeted approaches can screen many more putative regulatory elements with the same number of sgRNAs.

Three major CRISPR perturbation strategies were used: (1) small genetic perturbations induced by Cas9 nuclease (Cas9)^20,21^ and large genomic region deletions (~2–20 kilobases (kb)) induced with paired sgRNA^8,16,22^, (2) epigenetic repression, with deactivated Cas9 (dCas9) fused to a KRAB domain (CRISPR interference (CRISPRi))^23–25^, or (3) transcriptional activation, with dCas9 fused to activator domains (CRISPR activation (CRISPRa)^26–28^; Fig. 1a). All screens introduced sgRNAs into cells at low multiplicities of infection via lentiviral transduction followed by a bulk phenotyping method^9–12,14,16–18,22,29–31^. sgRNAs were then sequenced, and differences in sgRNA abundance were quantified to measure each sgRNA’s effect on the measured phenotype.

The ENCODE CRISPR screening database contains >540,000 individual perturbations covering 24.85 megabases (Mb; 0.82%) of the human genome (Methods). Regulatory activity was assayed for 56 genes and growth-related phenotypes in untreated and/or environmental perturbation contexts (for example, drug or stimulus) in 14 human cell lines, induced pluripotent stem cells (iPSCs) or iPSC-derived cell types, collectively identifying 865 distinct regions that significantly impacted a cellular phenotype when perturbed, hereafter referred to as CREs (Supplementary Tables 1 and 2 and Methods). In total, 4.0% (994,400/24,848,100) of perturbed bases displayed regulatory function, and 4.79% (2,547/53,197) of ENCODE SCREEN cCREs that were perturbed in at least one experiment directly overlapped a CRE. Notably, only 3.35% (29/865) of CREs did not directly overlap open chromatin regions, defined by DNase sequencing (DNase-seq) in 95 different cell and/or tissue types, or proximal enhancer-like signature cCREs (pELS) and distal enhancer-like signature (pDLS) cCREs, which demarcate accessible chromatin regions also marked by H3K27ac in at least one cell or tissue type; 99.7% of CREs (862/865) were within ±500 base pairs (bp) of these annotations

**Extended Data Fig. 1:**
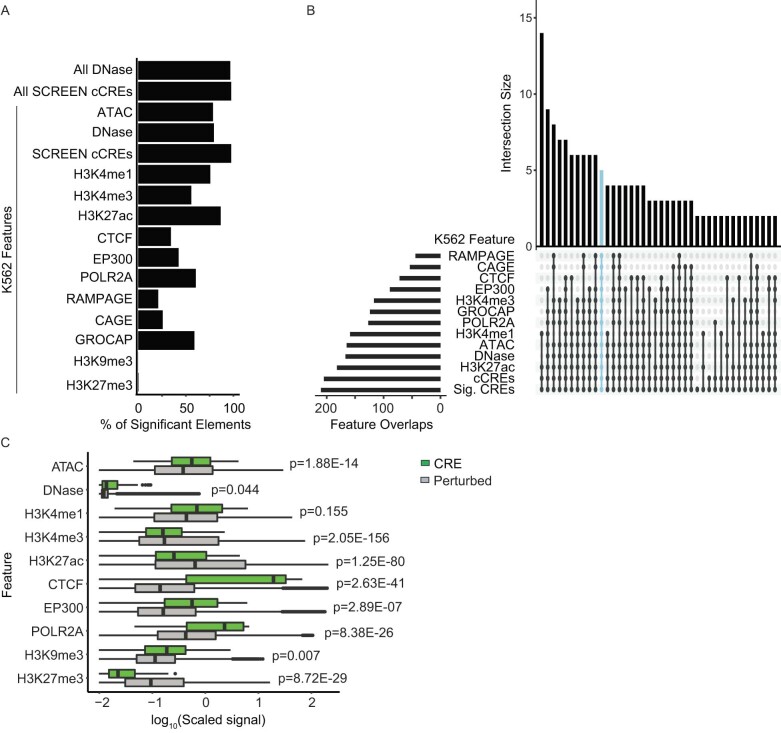
Integrated analysis of K562 screens nominates features of functional CREs. **A**) The percent of total significant CREs (n = 210) that intersect union sets of annotations from ENCODE biosamples and K562 annotations. **B**) Upset plot of the intersection of significant CREs with SCREEN K562 cCREs, and K562-annotated accessible chromatin regions, histone marks, EP300, CTCF, POLR2A, peaks. Blue highlight indicates CREs that intersect all features. **C**) Signal fold change over background for K562 features in CREs (n = 210 CREs, colored in green) versus perturbed regions (n = 3213 regions, colored in gray). Note each value was increased by 0.01 and then log_10_-transformed for visualization. All comparisons except H3K9me3 were significant at P value < 0.01 (Two-sided Wilcoxon test P values noted in the plot). Full test results and mean and median signal values reported in Supplementary Table 7. Each box ranges from the first quartile to the third quartile with a line drawn at the median. Lines extend to 1.5x the interquartile range and individual dots extending beyond this range indicate outliers.

Because most experiments were performed in K562 cells, we leveraged 53 noncoding CRISPR screens to gain insights into the characteristics and features that define CREs in this cellular context. Integrating these data, we found that 230.6 kb (2.82%) of the 8.2 Mb perturbed in greater than or equal to one experiment displayed control of gene expression or cellular growth (*n* = 355,356 unique perturbations; Fig. 1b, Supplementary Table 1 and Methods). Across all experiments, 0.49% of ENCODE SCREEN cCREs (11,447/2,348,854) intersected perturbed regions, and, of this subset, 5.31% (608/11,447) overlapped a functional hit CRE. We intersected the identified CREs (*n* = 210; Supplementary Table 4) with annotations of K562 cells and observed the greatest overlap with ENCODE SCREEN cCREs (97.6%, 205/210; two-sided Fisher’s exact test, *P* = 5.90 × 10^–10^, odds ratio (OR) = 7.88) and the greatest enrichment of H3K27ac, RNA polymerase II (RNA Pol II) and H3K4me3 peaks (OR = 22.1, 14.5 and 10.8, respectively, *P* < 1 × 10^–5^ for each; Fig. 1c and Supplementary Tables 5 and 6). Similar enrichments were observed for ENCODE SCREEN cCREs and the union set of DNase hypersensitive sites (DHSs) across 95 different cell and/or tissue types (Extended Data Fig. 1a and Supplementary Table 6). Together, these results suggest that many epigenetic and accessibility assays are largely indicative of regulatory activity in noncoding CRISPR screens.

We next interrogated which feature(s) best defined CREs identified in CRISPR screens. The vast majority of CREs in K562 cells overlapped either accessible chromatin regions or H3K27ac peaks (95.2%, 200/210; Extended Data Fig. 1b), in agreement with other cell lines (for example, HepG2, HCT116 and MCF-7)^32^. However, 24 CREs are marked by H3K27ac peaks but do not overlap DHSs, and 18 overlap DHSs but lack H3K27ac peaks (11.4% and 8.6%, respectively). Nine CREs lack either of these features in K562 cells, but seven of those elements are located within DHSs in at least one other ENCODE biosample. We observed a greater median signal for chromatin accessibility, H3K4me1, H3K9me3, EP300, POLR2A and CTCF at CREs (Extended Data Fig. 1c and Supplementary Table 7). Some exhibit different combinations of epigenomic features (Extended Data Fig. 1b), in agreement with previous enhancers identified in massively parallel reporter assay studies^33^.

**Extended Data Fig. 2:**
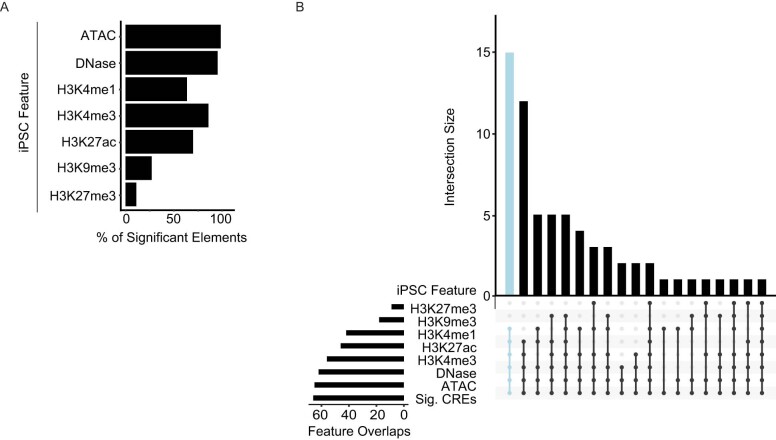
Features of functional CREs in iPSCs. **A**) Significant CREs (n = 66) that intersect union sets of annotations from WTC11 iPSCs, iPS DF6.9, and iPS DF19.11. **B**) Intersection of significant CREs with iPS-annotated accessible chromatin regions and histone mark peaks. Blue indicates CREs that intersect all activating annotations (H3K4me1/3, H3K27ac, ATAC, DNase).

To determine if these K562 CRE features were applicable in other cell types, we intersected CREs identified in nine additional cell types with assay for transposase-accessible chromatin with high-throughput sequencing *(*ATAC-seq), DNase-seq and H3K27ac chromatin immunoprecipitation with sequencing (ChIP–seq) peaks in the corresponding cell type (WTC11 iPSCs, *n* = 66 CREs; GM12878, *n* = 14 CREs; Jurkat, *n* = 12 CREs; A549, *n* = 4 CREs; HCT116, *n* = 3 CREs; MCF-7, *n* = 3 CREs; HepG2, *n* = 2 CREs; NCI-H460, *n* = 1 CREs; PC-3, *n* = 1 CREs). Across all cell types, the majority of CREs overlapped an accessible chromatin region, H3K27ac or both features (Fig. 1e and Supplementary Table 8). We then intersected the CREs in WTC11 iPSCs with additional activating and repressive histone mark ChIP–seq peaks and observed that most CREs overlapped regions with H3K4me1 and H3K4me3 in addition to H3K27ac, similar to the K562 CREs (Extended Data Fig. 2a). Interestingly, we also observed a greater proportion of CREs that overlap repressive histone marks (H3K9me3 and H3K27me3) in WTC11 iPSCs than in K562 cells and CREs that are marked by both active and repressive histone marks, consistent with the presence of poised and bivalent regulatory elements in stem cells^34–36^ (Extended Data Fig. 2a,b). Collectively, these results support accessible chromatin and/or H3K27ac as defining features of CREs but indicate potential cell-type specificities.

### CRISPR screen results are reproducible in validation experiments

To examine the reliability of the datasets, we compared the fold change (FC) in gene expression from individual sgRNA perturbations to the enrichment or depletion of those sgRNAs in CRISPR screens^9,10,12,17,37^. We found that the screen results significantly correlate with changes in mRNA expression of a CRE’s target gene in individual sgRNA validation experiments (*R*^2^ > 0.75 for all screens; Supplementary Fig. 1a–d and Supplementary Information Section 3).

**Extended Data Fig. 3:**
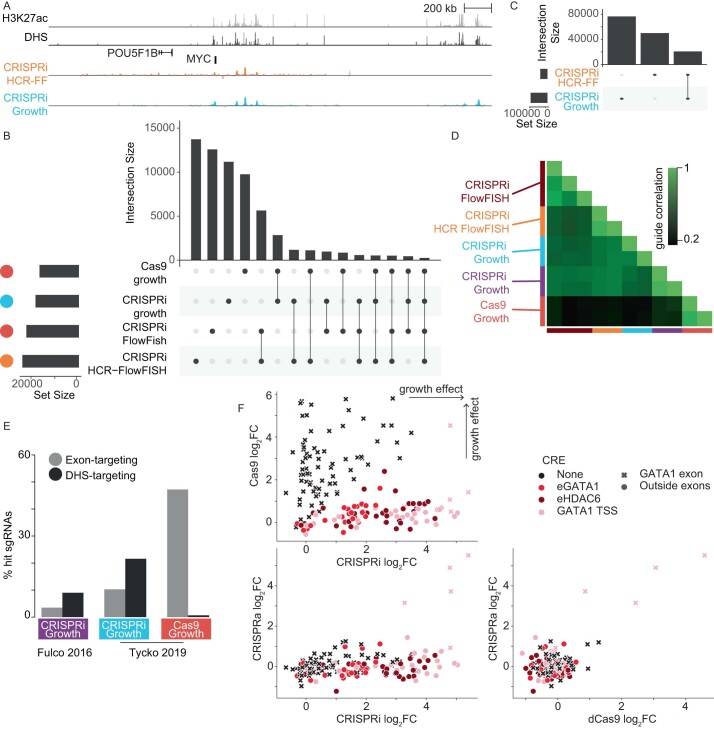
Overlapping targets and hits of CRISPR screens at the MYC and GATA1 loci. **A**) Genome browser snapshot of the *MYC* locus including H3K27ac (light gray) and DHS signal (dark gray) in K562 cells. CRISPR screen effects (mean log_2_FC, n = 2 screen replicates) and sgRNA locations (bars) for CRISPRi-HCR-FlowFISH (FF) (orange) and Tycko et al. 2019 (ref. ^17^) CRISPRi-growth (blue). **B**) Number of overlapping PAM coordinates across 5 screens in the *GATA1* and **C**) *MYC* loci. **D**) Pearson correlation for effects of sgRNAs that are shared across screens tiling *GATA1*. Each screen has 2-3 replicates shown as squares. **E**) Percentage of exon (gray, total n = 172, 78, 72 from left to right) or K562 DHS targeting guides in the *GATA1* locus chrX:48,773,708-48,801,225 (black, total n = 322, 153, 158 from left to right) with significantly high log_2_FC effect sizes (Z-test using mean and variance from negative controls p-value < 0.001). Note this is a conservative hit threshold, and some DHSs are not expected to affect GATA1 expression. **F**) Guide effects in *GATA1* tiling growth screens (Tycko et al. 2019 (ref. ^17^)) with different CRISPR modalities. Data is shown only for sgRNAs that target a previously-validated GATA1 CRE (colors) or a GATA1 exon (shape). Guides are filtered for high-specificity with GuideScan CFD > 0.2 (markers show mean log_2_FC, n = 2 screen replicates).

To interrogate how different screening approaches compared at the same CREs, we identified sgRNAs used multiple times across 16 screens with varied library sizes and designs at two commonly studied loci, *GATA1* (Fig. 1d) and *MYC* (Extended Data Fig. 3a–c). Together, these screens deployed >140,000 individual sgRNAs, perturbing 1,655 cCREs in *GATA1* and *MYC* flanking regions. For the 176 sgRNAs common between all five *GATA1* screens (after filtering with GuideScan^38,39^ cutting frequency determination (CFD) specificity scores of ≥0.2 to reduce possibly confounding off-target effects^17^), we observed strong replication within individual screening approaches (*n* = 5; Pearson correlation, minimum: 0.59, maximum: 0.90, mean: 0.77). For CRISPRi, there was strong correlation between experiments (*n* = 36; Pearson correlation, minimum: 0.42, maximum: 0.90, mean: 0.56), while we identified similar *MYC* CREs independent of phenotypic readout (Extended Data Fig. 3a). By contrast, there was low correlation between CRISPRi and Cas9 tiling at *GATA1* (*n* = 18; Pearson correlation, minimum: 0.15, maximum: 0.32, mean: 0.21; Extended Data Fig. 3d), with most significant Cas9 sgRNAs targeting exons and most significant CRISPRi sgRNAs targeting DHSs (Extended Data Fig. 3e,f). For CRISPRa, the only significant sgRNAs were directly at the transcription start site (TSS) and were shared with dCas9 alone, suggesting dCas9-mediated steric hindrance effects (Extended Data Fig. 3f). Cas9 and dCas9 alone can map functional motifs with finer resolution^11,40^, but some CRISPRi-responsive enhancers are not affected by sgRNA perturbations with these modalities (for example, the *GATA1* enhancers)^17^. CRISPRa can be used in distinct contexts to find enhancers^18,30^ or long noncoding RNAs^41^ but has not yet been as widely adopted for noncoding screens, and more data are needed to inform guidelines for its use.

### Integrated CRISPR screen analysis informs design guidelines

**Extended Data Fig. 4:**
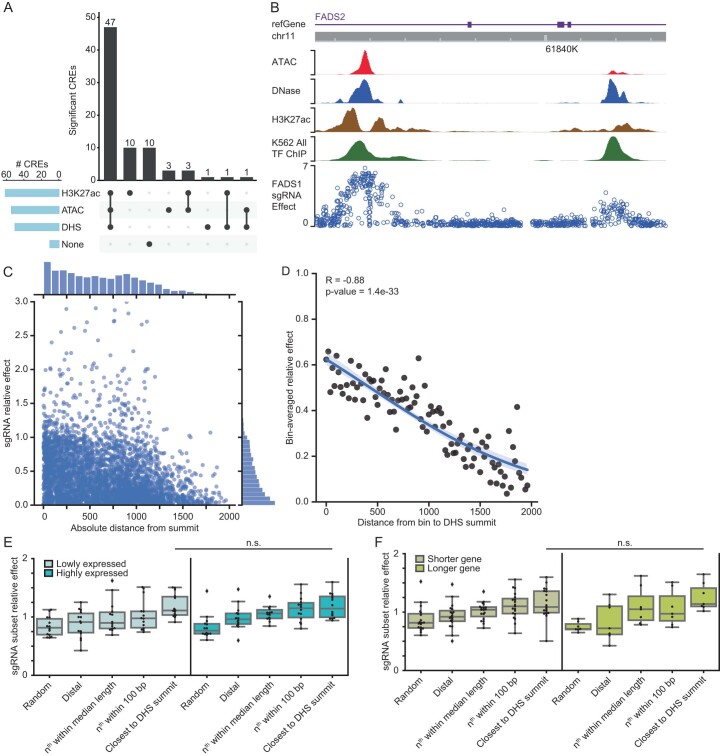
Selecting cCREs and targeting sgRNAs near DHS summits. **A**) Epigenetic feature peak intersections with significant CREs identified in 16 HCR-FlowFISH screens. **B**) Browser track highlighting two significant enhancers within *FADS2*. The K562 All TF ChIP track was created by concatenating all ENCODE K562 TF ChIP-seq experiments, and de-duplicating non-unique peak calls. The height of the track represents the number of unique TFs with peaks at a position. The average effects of each sgRNA from the *FADS1* HCR-FlowFISH screen (n = 2 replicates). **C**) The effects of all sgRNAs across all HCR-FlowFISH screens within 2000 bases of a significant enhancer’s DHS peak are plotted, normalized to the average effect of all sgRNAs in their enhancer. **D**) Same as (**C**), except sgRNAs are separated into 20 bp bins, with the mean of the sgRNA’s enhancer-relative effects plotted for each bin; loess regression line drawn in blue. **E**) Comparison of sgRNA selection strategies for K562 HCR FlowFISH gene screens (n = 20 loci), separated by gene expression levels (lowly expressed ≤ 100 TPM, highly expressed > 100 TPM) or **F)** gene body lengths (shorter gene ≤ 20 kb, longer gene > 20 kb) or. Points reflect the effects of 10 sgRNAs for significant enhancers, normalized to the mean effect of all sgRNAs in that enhancer. ‘Random’ is the average of 100 random subsets from across the DHS peak. ‘Distal’ are sgRNAs closest to half the median DHS peak length (179 bp) from the summit. Every ‘n^th^’ sgRNA is selected by arranging sgRNAs in order of their PAM’s genomic coordinate, and selecting every n^th^ sgRNA such that their ranked orders are evenly spaced. ‘Closest’ sgRNAs are nearest to the DHS summit. Boxes show the quartiles, with a line at the median, lines extend to 1.5 times the interquartile range, and dots beyond lines show outliers. Significance evaluated using Welch’s t-test on each pairwise comparison.

To improve sgRNA selection for noncoding CRISPRi screens to balance scale, sensitivity and practicality, we analyzed 15 highly sensitive CRISPRi hybridization chain reaction–fluorescence in situ hybridization coupled with flow cytometry (CRISPRi HCR–FlowFISH) screens designed with unbiased tiling over 100 kb at eight loci in K562 cells^8–10,16^. Consistent with our findings described earlier, the significant CREs were found in accessible chromatin (74%) or H3K27ac ChIP–seq peaks (80%), with the majority having both epigenetic features (Extended Data Fig. 4a). Thus, a combination of CRE-associated epigenetic features (Extended Data Fig. 1b) can be used to nominate cCRE targets.

**Fig. 2:**
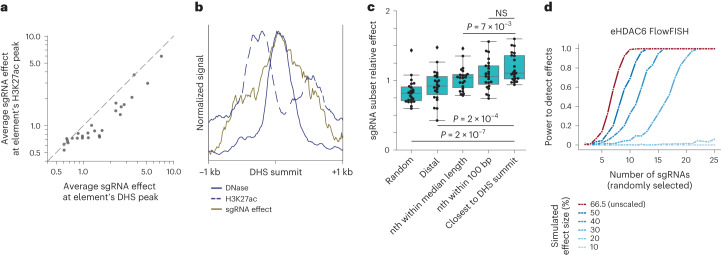
Integrated analysis of noncoding CRISPR screens provides guidelines for selecting cCRE targets and sgRNAs. **a**, Average effects of all sgRNAs within DHS or H3K27ac peaks at significant enhancers intersecting both epigenetic features. **b**, bigWig *P* value signal tracks for H3K27ac ChIP–seq and DNase-seq and bp-normalized effects of 6,338 sgRNAs within ±1 kb of DHS summits for 27 significant enhancers intersecting 32 DHS and H3K27ac peaks (*n* = 20 loci from HCR–FlowFish screens). **c**, Comparison of sgRNA selection strategies. Points reflect effects of ten sgRNAs selected by the indicated method for significant enhancers normalized to the mean effect of all sgRNAs in that enhancer. ‘Random’ is the average of 100 random subsets across the DHS peak. ‘Distal’ are sgRNAs closest to half the median DHS peak length (179 bp) from the summit. Every ‘nth’ sgRNA is selected by ordering sgRNAs by their protospacer-adjacent motif’s (PAM’s) genomic coordinate and selecting every nth sgRNA such that their ranked orders are evenly spaced. ‘Closest’ sgRNAs are nearest to the DHS summit. Boxes show quartiles, with lines at medians; lines extend 1.5 times the interquartile range. Significance was evaluated using a Welch’s *t*-test on the indicated pairwise comparisons; NS, not significant. **d**, Power simulation to detect significant effects on *GATA1* expression as a function of enhancer effect sizes and sgRNA number. Power was computed by simulations of CRISPRi FlowFISH data, where sgRNA effects in the eHDAC6 element were scaled such that the average adjusted effect of all sgRNAs in the enhancer was 10–50% or unscaled (*n* = 3 biological replicates).

**Extended Data Fig. 5:**
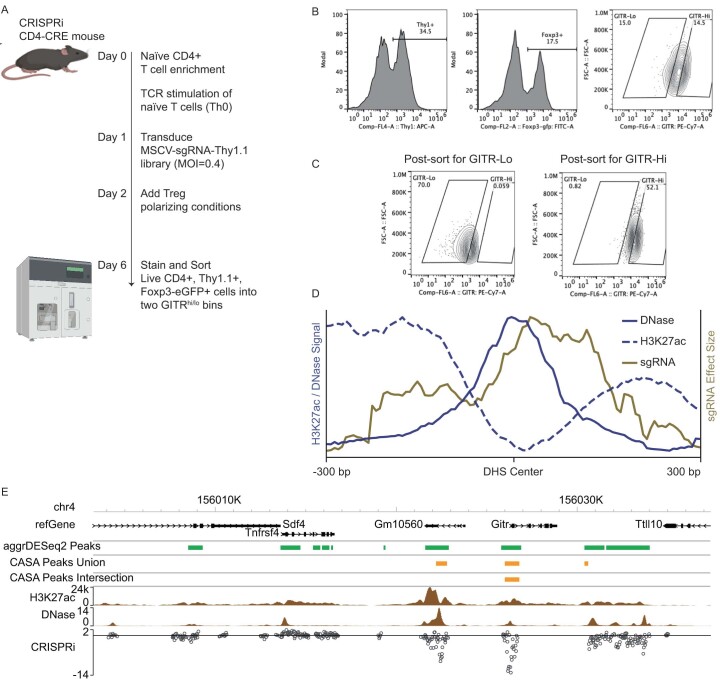
A stain-and-sort screen for GITR expression in primary mouse Regulatory T-cells. **A**) Schematic for a screen for GITR expression in primary mouse Regulatory T-cells. The sgRNA library is delivered by retrovirus that also contains a Thy1.1 surface marker reporter gene. **B**) Gates used for sorting viable CD4 + /Thy1 + /Foxp3-eGFP+ cells into GITR-Lo and GITR-Hi bins. **C**) Flow analysis of GITR expression in the sorted populations. **D**) Correlation between accessibility score and sgRNA perturbation effect. **E**) Genome browser view of GITR locus and sgRNA effects (circles show mean of n = 4 screen biological replicates). The union and intersection of CASA peaks across replicates and the aggrDESeq2 peak calls are shown in orange and green, respectively.

Optimizing cCRE-targeting sgRNAs is crucial for maximizing perturbation strength without compromising practicality or scale. We compared relative sgRNA perturbation effects within significant enhancers and observed that sgRNAs overlapping a DHS peak induced stronger perturbations than those overlapping H3K27ac peaks (Fig. 2a; binomial test *P* < 0.001). Further, sgRNA effects across these enhancers revealed local perturbation maxima near the enhancers’ DHS summits (Fig. 2b and Extended Data Fig. 4b–d). Aggregating all significant enhancers together, we found that sgRNA effects are strongest nearest the DHS summit, with a near-linear decrease as a function of distance from the summit (Fig. 2b and Extended Data Fig. 4c,d). This result held regardless of gene expression level or length (*n* = 20 loci; Extended Data Fig. 4e,f). We compared methods for selecting sgRNA subsets and confirmed that sgRNAs closest to the DHS summit performed better than sgRNAs that were farther away or randomly or evenly spaced apart (Fig. 2c). This selection method is straightforward and only requires summit calls, standard output from peak callers such as MACS2 (ref. ^42^). To validate these findings in an orthogonal biological context, we performed a CRISPRi screen in primary mouse regulatory T cells by staining and sorting for GITR expression and found a similar relationship with stronger perturbation effects closer to DHS summits than H3K27ac summits (Extended Data Fig. 5a–e).

**Extended Data Fig. 6:**
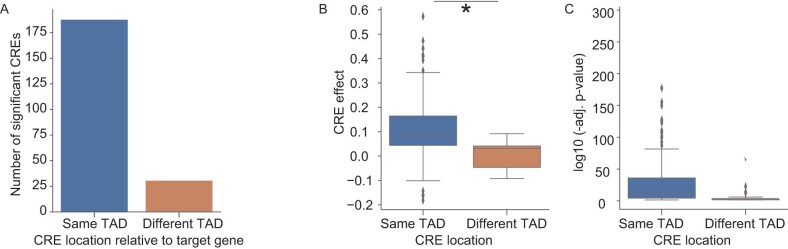
The majority of and the strongest significant CREs are within the same TAD as their target gene. **A**) Significant CREs in K562 screens with adjusted p-values ≤ 0.05 that reside inside a K562 HiC TAD were included for analysis. For each CRE’s target gene, it was determined if the consensus RefSeq promoter 1 kb window around the TSS was in the same TAD as the CRE. **B**) The effect size of the CREs in the same or different TAD as the target gene (n = 188 and 31, respectively; * denotes p = 2.8e-12 by Welch’s t-test). **C**) The p-values for these CREs.

As enhancers can be far from their target gene, screening all potential cCREs in this range may not be feasible^12,43,44^. When considering all K562 screens, we found that 86% of significant CREs are within the same TADs as their target gene and had greater effect sizes than those in different TADs (Extended Data Fig. 6a–c). Predictive modeling using the activity-by-contact (ABC) model^12,43^ identified 43% of these CREs. Together, chromatin contact maps and predictive modeling can be used to prioritize target cCREs in a screen.

**Extended Data Fig. 7:**
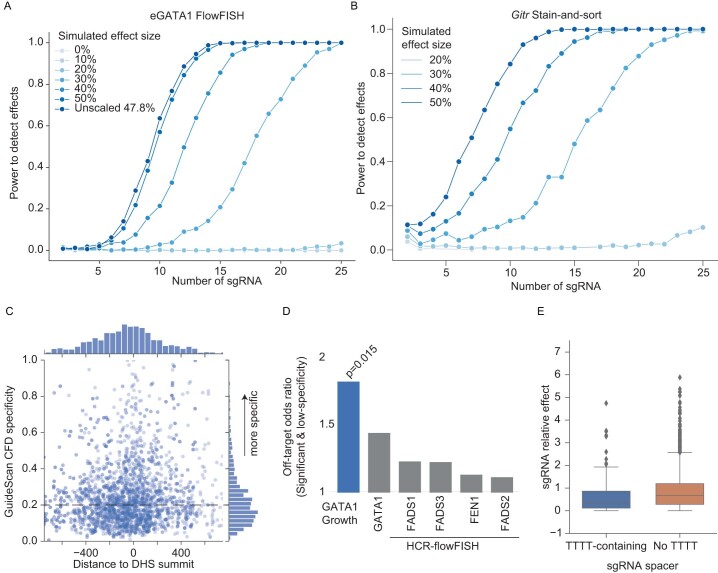
Power related to sgRNAs per element and impact of sgRNA specificity and sequence. **A**) The power to detect significant effects on gene expression as a function of the number of sgRNAs targeting each element and the effect size of that element. Power was computed by simulations based on the average sgRNA effects from three biological replicates of GATA1 CRISPRi-FlowFISH data, where the individual sgRNA effects in the eGATA1 element were scaled such that the average adjusted effect of all sgRNAs in the enhancer was 10-50% of the promoter, in steps of 10%. **B**) Power analysis for detecting significant effects as a function of the number of sgRNAs targeting the *Gitr* enhancer chr4:156021490-156022916. Simulations were based on the average sgRNA effects from four biological replicates of the GITR-staining Sort-seq experiment. **C**) sgRNA PAM distance to DHS summits compared with GuideScan CFD specificity scores, for all GuideScan sgRNAs in HCR-FlowFISH identified CREs that intersect DHS and H3K27ac peaks. Horizontal dashed line indicates GuideScan CFD specificity threshold of 0.2. **D**) Enrichment of sgRNAs with significant effects among sgRNAs with low specificity scores (GuideScan CFD < 0.2) in regions at least 1 kilobase away from any DHS peak in K562 cells for the indicated screens. The p-value from Fisher’s exact test is shown for each, and the significant (p < 0.05) bar is colored. **E**) Distribution of sgRNA effects normalized to the average effect of all sgRNAs in their respective CREs, for sgRNAs with spacers that do or do not contain a ‘TTTT’ U6 termination sequence, using sgRNAs that target significant enhancers that intersect DHS and H3K27ac peaks. Boxes show the quartiles, with a line at the median, lines extend to 1.5 times the interquartile range, and dots show outliers. TTTT-containing sgRNA n = 195; Non TTTT-containing sgRNA n = 3940 (Welch’s t-test P value = 1.7e-4).

Next, we investigated the minimally sufficient number of sgRNAs needed to test a target’s significance at a given effect size. We analyzed a *GATA1* FlowFISH screen^10^ and observed that 13 sgRNAs, selected randomly within the eHDAC6 enhancer, are required to provide over 80% power to detect enhancers with a 40% or greater effect on gene expression (Fig. 2d). We found similar results for eGATA1 and mouse regulatory T cell *Tnfrsf18* (*Gitr*) enhancers (Extended Data Figs. 5e and 7a,b).

sgRNA specificity and sequence filters display different impacts between gene expression and proliferation-based screens. Low-specificity sgRNAs often confound proliferation-based screens due to off-target toxicity^17^. A GuideScan aggregated CFD specificity score of ≥0.2 is an effective filter, and several high CFD score sgRNAs typically remain near the DHS peak (Extended Data Fig. 7c)^45^. However, we found that significant sgRNAs in HCR–FlowFISH screens were not significantly enriched for low-specificity sgRNAs (Extended Data Fig. 7d). Therefore, specificity filters as stringent as a GuideScan aggregated CFD specificity score of ≥0.2 may not be needed to avoid false positives in HCR–FlowFISH screens, in contrast to growth screens. sgRNA spacer sequence also affects efficacy; sgRNAs containing the U6 promoter termination sequence (‘TTTT’)^46^ had reduced relative effect sizes (Extended Data Fig. 7e; Welch’s *t*-test *P* = 1.7 × 10^–4^).

**Extended Data Fig. 8:**
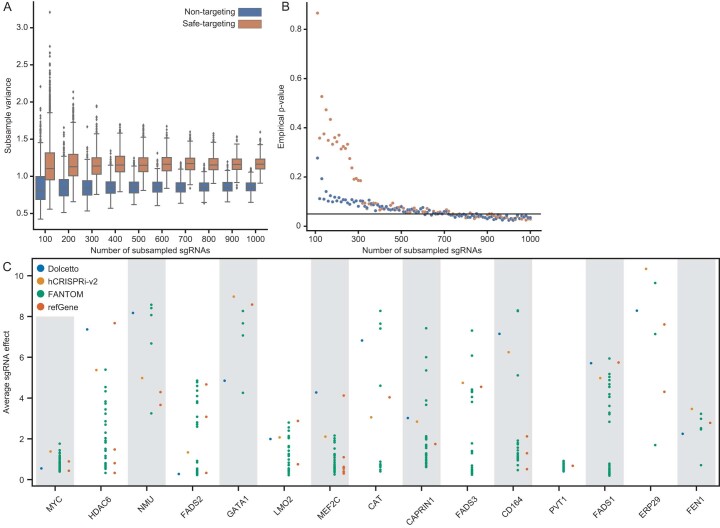
Evaluating methods of selecting negative and positive control sgRNAs. **A**) Boxplot of subsample variances for negative control sgRNAs in the CD164 HCR-FlowFISH screen, in increments of 100 sgRNAs subsampled 1000 times each from a total of 1000 sgRNAs for each type of negative control sgRNA. Each type of negative control was subsampled separately. Boxes show the quartiles, with a line at the median, lines extend to 1.5 times the interquartile range, and dots show outliers. **B**) Empirical P values from Levene’s test on subsampled negative control sgRNAs, in increments of 10 sgRNAs subsampled 1000 times, compared to the entire set of the respective type of negative control sgRNA. P = 0.05 threshold is indicated by the black line. **C**) Comparison of the average effect from both biological replicates of the 10 sgRNAs closest to the FANTOM5- and refGene-nominated TSSs for the HCR-FlowFISH genes against the sgRNAs provided by the Dolcetto or the hCRISPRi-v2 libraries, which may target one or more of these–or distinct–TSSs. Each point reflects an individual TSS (for the FANTOM5 and refGene TSSs) or the set of 4-10 sgRNAs from the Dolcetto or hCRISPRi-v2 libraries that were tested in the HCR-FlowFISH screens.

Negative-control sgRNAs are necessary to calibrate the null phenotype and test significance. Screens use either nontargeting sgRNAs or safe-targeting sgRNAs^47^ at inactive loci. Previous growth screens suggest that safe-targeting sgRNAs have stronger effects than nontargeting sgRNAs due to DNA damage effects^47^. By contrast, there was no significant difference in the average effect of nontargeting versus safe-targeting sgRNAs in CRISPRi HCR–FlowFISH screens using 1,000 of both types of negative controls (Welch’s *t*-test *P* = 0.23; Supplementary Table 9). However, safe-targeting sgRNAs had significantly greater variance, demonstrating that they are more stringent controls for significance testing (Extended Data Fig. 8a; safe-targeting variance = 1.17 or nontargeting = 0.86, Levene’s test *P* < 0.001). Although increasing the number of control sgRNAs reduces their variance, there was no statistically significant difference in the variance of 700 safe-targeting controls compared to all 1,000, suggesting that this may be sufficient for large-scale screens (Extended Data Fig. 8b). To facilitate direct comparisons across screens, we provide a common set of safe-targeting sgRNAs (Supplementary Table 10)^47^. We note that these safe-targeting sgRNAs were designed based on existing Roadmap Epigenomic data and may inadvertently target active loci in a novel cell type or sample.

Finally, sufficient numbers of sgRNAs targeting the measured gene’s promoter should be included as positive controls to ensure that strong perturbations can be sensitively detected and to estimate the upper bound of measurable effect sizes^47–49^. We compared the average effects of the ten sgRNAs closest to each FANTOM and RefGene TSS for the HCR–FlowFISH genes, along with the four to ten sgRNAs from the human CRISPRi Dolcetto^49^ or hCRISPRi-v2 (ref. ^48^) libraries that were included in our libraries. We found that sgRNAs from the Dolcetto or hCRISPRi-v2 libraries provided average effects similar to the maximum average effect from perturbing all of the FANTOM and/or RefGene TSS(s) for 12 of 14 genes (Extended Data Fig. 8c). However, for *FADS2*, there were greater than twofold larger effects at some FANTOM and RefGene TSS(s) than the published sgRNAs. Because neither Dolcetto nor hCRISPRi-v2 was consistently best, including sgRNAs from both published libraries increases the likelihood of having potent positive controls, but designing ten sgRNAs nearest every TSS (where space allows) maximizes it.

To facilitate sgRNA library design in accordance with these recommendations, we provide a summary of common sgRNA design tools (Supplementary Table 11). As a resource, we used GuideScan2 (ref. ^38^) to design sgRNA sets with and without filters for all human and mouse ENCODE SCREEN^6^ cCREs (Supplementary Fig. 2, Supplementary Table 8 and Supplementary Section 4). These sets include at least ten sgRNAs for targeting 85% and 60% (without and with filters, respectively) of the 249,464 human proximal enhancer-like cCREs and 86% and 70% of the 111,218 in mice^50^. Importantly, these design guidelines are based on modeling of data produced from experiments that were conducted at similar coverage and power, deviations from which may require including additional control or targeting sgRNAs.

### Cell and sequencing coverage impact CRE and sgRNA detection

**Fig. 3:**
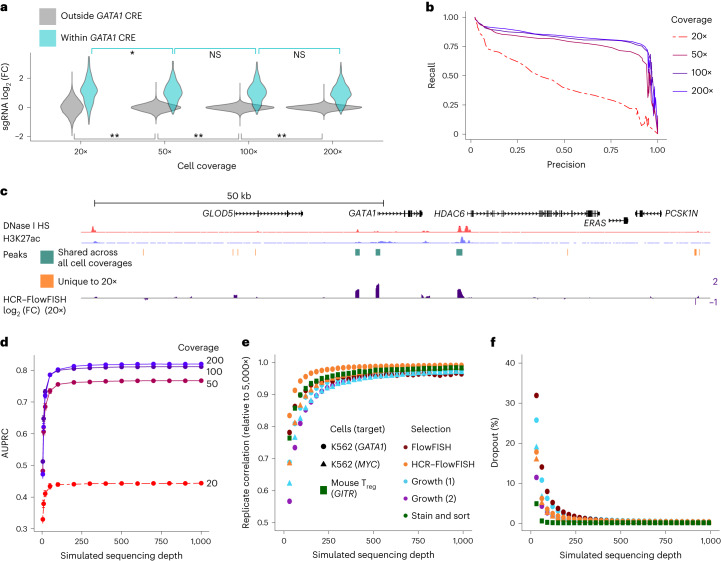
Cell coverage and sequencing depth impact reliable detection of CREs. **a**, Distributions of HCR–FlowFish guidewise log_2_ (FC) effect sizes (total of 13,732 PAMs targeted) at various cell coverages separately for sgRNA targets within (*N* = 288) and outside known *GATA1* CREs (*n* = 13,444). Asterisks denote significant changes in variance; *P ≤ 0.01 and **P < 2.2 × 10^–16^ by two-sided Levene’s test; NS, *P* > 0.2. **b**, Precision–recall curve for identifying *GATA1* CRE-targeting sgRNAs using effect sizes from various cell coverages (AUPRC: 20× = 0.44, 50× = 0.77, 100× = 0.81, 200× = 0.82; CRISPRi HCR–FlowFish). **c**, log_2_ (FC) signals for 20× and CASA peak calls shared across all coverages and unique to 20×. DNaseI HS, DNase I hypersensitive site. **d**, AUPRC for identifying *GATA1* CRE-targeting sgRNAs with varying sequencing depth (bootstrap sampled) and cell coverages (20×, 50×, 100× and 200×). Dots and error bars indicate averages and 99% confidence intervals over ten bootstrap samples. **e**,**f**, Biological replicate reproducibility (Pearson correlation of guidewise log_2_ (FC)) normalized to 5,000× simulated sequencing depth (**e**) and guide dropout rate (dropout defined as less than ten mapped reads) in diverse CRISPRi screens with varying sequencing depth (bootstrap sampled; **f**). Dots show an average over 100 bootstrap samples. The *GATA1* (circles) and *MYC* (triangles) screens in human K562 cells were performed with varied readout methods (colors). The GITR screen (rectangle) in mouse regulatory T cells (T_reg_) used protein staining followed by sorting. The growth datasets included are (1) Tycko et al.^17^ and (2) Fulco et al.^12^.

We next interrogated how varying the number of cells per sgRNA impacts accuracy of CRE identification by using CRISPRi HCR–FlowFISH experiments at the *GATA1* locus (Methods and Supplementary Table 12). We tested whether positive sgRNAs (those targeting the three validated CREs; *n* = 288) can be distinguished from negative sgRNAs (outside the three CREs; *n* = 13,444) by their log_2_ (FC) effect sizes. At low cell coverage (20×), effect sizes of both sets of sgRNAs had high variance, leading to limited statistical power for distinguishing positive signals from negative-control background (Fig. 3a). With increasing cell coverage, the variance of negative sgRNAs approaches 0, whereas the variance of positive sgRNAs stabilizes for coverages ≥50×. Thus, increasing cell coverage led to higher precision and sensitivity for distinguishing positive from negative sgRNAs (area under precision recall curve (AUPRC): 20× = 0.44, 50× = 0.77, 100× = 0.81, 200× = 0.82; CRISPRi HCR–FlowFish; Fig. 3b). Further, CASA peak calling with 50–200× cell coverage resulted in accurate identification of the known *GATA1* CREs, whereas the 20× data resulted in spurious CRE calls lacking CRE-associated epigenetic marks (Fig. 3c). Last, with cell coverage of 20×, we observed a high dropout rate (sgRNAs with less than ten mapped reads in low- or high-expression sorting bins) of ~12%, which decreases to less than 1% with cell coverage greater than 50× (Supplementary Fig. 3). Based on these strong-to-moderate *GATA1* CREs, experimental cell coverage of at least 100× should be considered the minimum, although higher coverage is advised when feasible. For example, coverage as high as 11,000× has been used in noncoding growth-based screens^17^.

**Extended Data Fig. 9:**
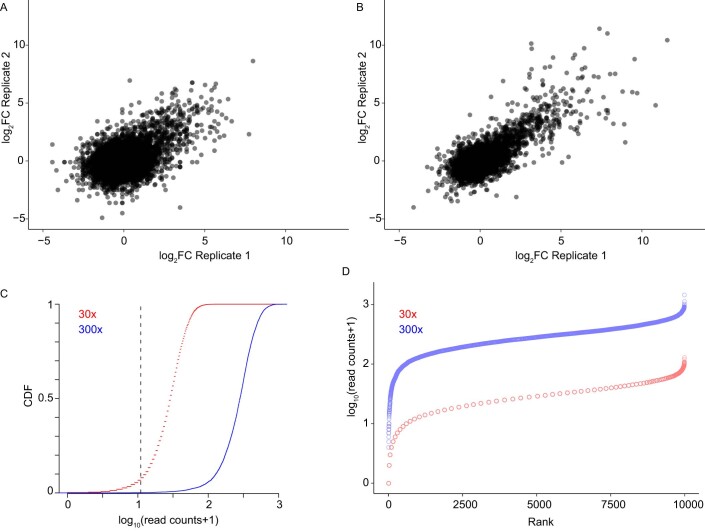
Representative bootstrap samples for low and high sequencing depths using K562 GATA1 locus CRISPRi growth screen. **A**) Biological replicate 1 log_2_FC vs Biological replicate 2 log_2_FC (Z-score) for 30x bootstrapped sequencing depth (9977 sgRNAs, R = 0.45). **B**) Biological replicate 1 log_2_FC vs Biological replicate 2 log_2_FC for 300x (R = 0.73). **C)** Empirical cumulative distribution function of sgRNA read counts across the library for samples at 30x (red) or 300x (blue) bootstrapped sequencing depth (vertical dashed line: read count = 10). **D**) Dropout plot showing sgRNAs ranked by read counts at 30x (red) and 300x (blue) bootstrapped sequencing depth.

We also sought to derive sequencing depth guidelines for noncoding CRISPR screens. We sampled, on average, 5× to 1,000× sequencing reads per sgRNA and found that with 250× sequencing depth or higher, accuracy of HCR–FlowFISH screens for *GATA1* CREs is limited by cell coverage, such that further increases in sequencing depth only marginally improves accuracy (Fig. 3d). We repeated the analysis in five other CRISPR screens, including growth screens performed at *GATA1* and *MYC* loci, and found that 250× sequencing depth was a reasonable minimum for CRE identification accuracy. Further, we observed saturation of biological replicate correlation of guide effects and of guide dropout rate starting at 250× sequencing depth (replicate normalized log_2_ (FC) *R* > 0.9 and average dropout rate of <2% for all screens; Fig. 3e,f and Extended Data Fig. 9). In addition, we assessed normalization strategies and found that mean-normalized effect size calculations were more reproducible between biological replicates than linear-transformed effects. This finding was consistent for *GATA1* screens with varied phenotyping strategies (Supplementary Fig. 4a) and for HCR–FlowFISH screens across 20 loci (Supplementary Fig. 4b).

### CASA provides more conservative CRE calls than other methods

**Fig. 4:**
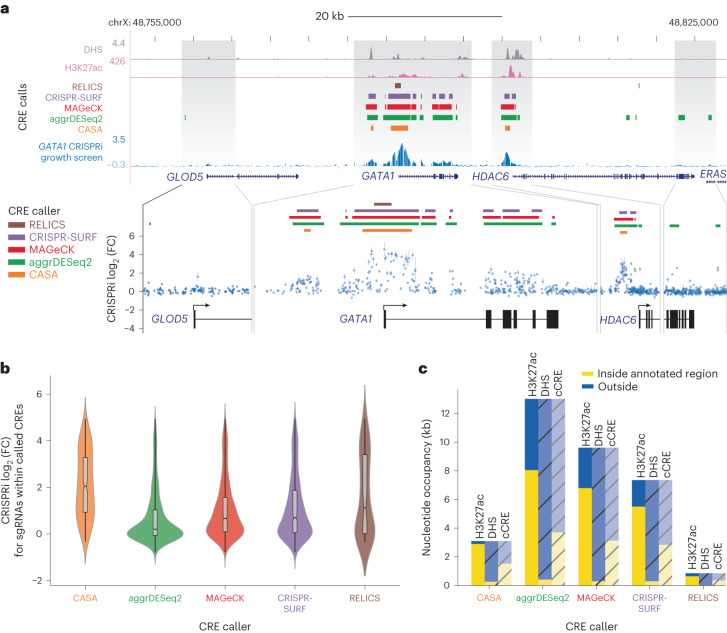
CRISPR screen analysis tools identify CREs with varying selectivity. **a**, sgRNA-mediated growth effects (blue), H3K27ac ChIP signal (pink) and DHS (gray) for a CRISPRi growth screen at the *GATA1* locus. sgRNAs were filtered to remove any low-specificity sgRNAs (GuideScan aggregated CFD < 0.2), which could cause confounding off-target toxicities. Dense tracks show peak calls using five different CRISPR screen analysis tools: CASA (orange), aggrDESeq2 (green), MAGeCK (red), CRISPR-SURF (purple) and RELICS (brown). Zoomed-in regions show log_2_ (FC) of individual sgRNA effects (points indicate the mean values, and bars indicate the minimum–maximum range of observations between *n* = 2 replicates). **b**, Distribution of average guide effects calculated from two experimental replicates for sgRNAs falling within peaks identified by different CRISPR screen analysis tools (center line, median; notch, confidence interval of the median; box limits, first and third quartiles; whiskers, range of all data points; violin, kernel density estimation; *n* = 204, 1,218, 715, 623 and 71 sgRNAs within CREs from left to right; Welch’s two-tailed *t*-test versus shuffled –log_10_ (*P*) = 55.2, 59.3, 68.8, 66.6 and 8.3). **c**, CRISPRi screen peak area intersecting (yellow) and complementing (blue) annotated chromatin features (H3K27ac, DHS) and ENCODE SCREEN cCREs. Shading and hashing indicate which reference annotation is used for the comparison, and total bar height reflects total genomic area demarcated as significant by the peak caller.

Noncoding CRISPR screens can produce noisy results when sgRNAs generate variable effects in a genomic interval (Fig. 4a). Multiple analysis approaches, or ‘peak callers’, aggregate individual sgRNA measurements from dense tiling screens to nominate CREs. We investigated the use of five peak callers: element-level aggregation of DESeq2 (aggrDESeq2), CASA, CRISPR-SURF, MAGeCK and RELICS^9,51–54^ (Supplementary Table 13). We benchmarked the identification of *GATA1* CREs using a CRISPRi tiling growth screen, excluding low-specificity sgRNAs (Fig. 4). Although a comprehensive, fully validated ground truth CRE set is lacking, these CREs have been rigorously epigenetically profiled and studied across multiple functional characterization assays^9,10,12,15^.

All peak callers nominated the promoter for *GATA1* (Fig. 4a) as a CRE. Additionally, CREs called by all five methods corresponded with significantly higher sgRNA effects than shuffled control elements (Fig. 4b; *P* ≤ 5 × 10^–9^, Welch’s two-tailed *t*-test). However, the total number of CREs varied across each method, with aggrDESeq2 identifying the most (*n* = 21) and CASA and RELICS identifying the least (*n* = 3). Meanwhile, peaks called by CASA, CRISPR-SURF and MaGeCK had the greatest proportional overlap with annotated ENCODE SCREEN cCREs, H3K27ac peaks and DHSs (Fig. 4c). aggrDESeq2 CREs yielded the largest total overlap but also identified a greater proportion of CREs outside of annotations. We found that canonical *GATA1* elements are most similar to CASA and RELICS CREs and least similar to aggrDESeq2 CREs (Supplementary Fig. 5a). Finally, we inspected the intersection of *GATA1* CRE calls from each method and found that CASA was the only peak calling method that lacked unique *GATA1* CRE calls (Supplementary Fig. 5b).

To determine each method’s susceptibility to potential sgRNA off-target effects, we reanalyzed the *GATA1* screen with low-specificity sgRNAs included (Methods and Supplementary Fig. 6a–d). The total number of CREs called by aggrDESeq2 increased by more than threefold (21 CREs versus 68 CREs). The total number of CREs called by CRISPR-SURF, MAGeCK and RELICS increased by 12, 4 and 2, respectively, whereas the number of CREs identified by CASA did not change. After removing the single most significant sgRNA per bin, the total number of aggrDESeq2 peak calls decreased to 11, indicating that the method is sensitive to potential outliers. Collectively, these results support CASA as the preferred method for CRE calling. To facilitate future analytical development and benchmarking, we propose processed data file formats that capture critical experimental parameters and include sgRNA-level and CRE-level effect quantification (Supplementary Information Sections 5 and 6).

### Perturbation dynamics affect screen sensitivity

**Fig. 5:**
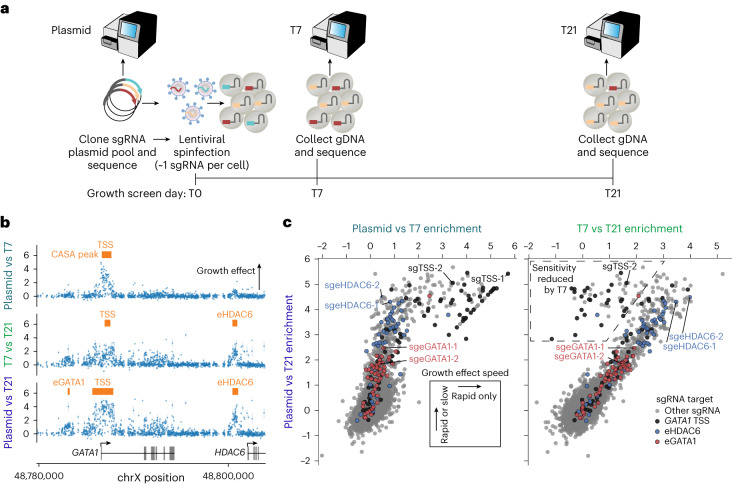
Perturbation dynamics impact screen sensitivity and resolution. **a**, Timeline of CRISPRi growth screen with quantified sgRNA abundances of the sgRNA plasmid library before delivery and at T7 and T21 after sgRNA lentiviral delivery. **b**, CRISPRi growth screen at the *GATA1* locus shown with different time point comparisons (top, plasmid versus T7; middle, T7 versus T21; bottom, plasmid versus T21) used to compute sgRNA effect sizes. Each dot shows the average log_2_ (FC) effect size of two biological replicates for an sgRNA, and the error bar shows the range. CASA peak calls for significant growth effects are shown. The *GATA1*-regulating CREs eGATA1, *GATA1* TSS and eHDAC6 are labeled with their corresponding CASA peak calls. **c**, Scatter plot of sgRNA effect sizes as determined by different time point comparisons. Each dot shows the average of two biological replicates for an sgRNA. Black or colored dots are sgRNAs targeting the TSS or enhancers, respectively. The sgRNAs along the diagonal line of points, including sgTSS-1, drop out by T7 and thus are absent from the T7 versus T21 comparison. sgRNAs selected for validation assays are labeled.

Our integrated dataset provides an opportunity to investigate possible interactions between perturbation timing, sgRNA effect sizes and phenotyping strategy. Conceptually, a higher-effect-size sgRNA would be expected to display detectable phenotypic impacts sooner than a weaker-effect-size sgRNA, but there is no clear consensus on if the initial plasmid pool of sgRNAs or an early time point after lentiviral delivery is the best initial sample comparator to identify sgRNA effects. We leveraged multiple *GATA1* CRISPRi growth screen time points and sequenced sgRNAs in the predelivery plasmid pool, at 7 days after lentiviral guide delivery to cells (T7) and at an end point after 21 days (T21; Fig. 5a). Comparing plasmid to T7, we observed a significant CRE at the promoter but did not identify the distal eGATA1 and eHDAC6 CREs (Fig. 5b). However, both distal CREs were identified in the plasmid–T21 or T7–T21 comparison (Fig. 5b), and the peak at the promoter widened by ~1 kb with increasing sgRNA effect sizes.

Although the sgRNA effect sizes from these two time point comparisons are correlated (*R*^2^ = 0.71), a subset of sgRNAs (<1%) displayed time point-dependent effects (Fig. 5c). These sgRNAs are strong (log_2_ (FC) > 3) in a plasmid–T21 comparison but have reduced effect sizes in a T7–T21 comparison. These sgRNAs largely target the *GATA1* TSS. One of these sgRNAs (sgTSS-2) was individually validated to reduce *GATA1* expression and growth (Supplementary Fig. 1d and Supplementary Table 14). Another validated sgRNA (sgTSS-1, Supplementary Fig. 1d) displayed the third strongest effect in the plasmid–T21 comparison (log_2_ (FC) = 5.4) and the strongest effect in the plasmid–T7 comparison (log_2_ (FC) = 5.7) but dropped out by T7 and was not observed in the T7–T21 comparison and thus became a false negative. Together, this suggests that these rapidly depleted sgRNAs can cause bonafide growth phenotypes, and the strongest hits may be most affected by reduced sensitivity in the T7–T21 comparison.

We reasoned that screens based on growth may be more sensitive to perturbation dynamics than screens that directly read out transcriptional changes. Indeed, an HCR–FlowFISH screen of *GATA1*, in which sgRNA abundances were compared before and 2 days after CRISPRi induction by doxycycline, identified both the promoter and the two distal CREs (Fig. 1d). This screen format was not susceptible to reduced power to detect the strongest TSS-targeting sgRNAs. Together, we suggest comparisons to initial sgRNA abundance before starting phenotypic selection, for example, by measuring sgRNA abundance in the input plasmid library or in cells before CRISPRi expression in an inducible system.

### CRISPRi effects in the gene body are strand specific

**Fig. 6:**
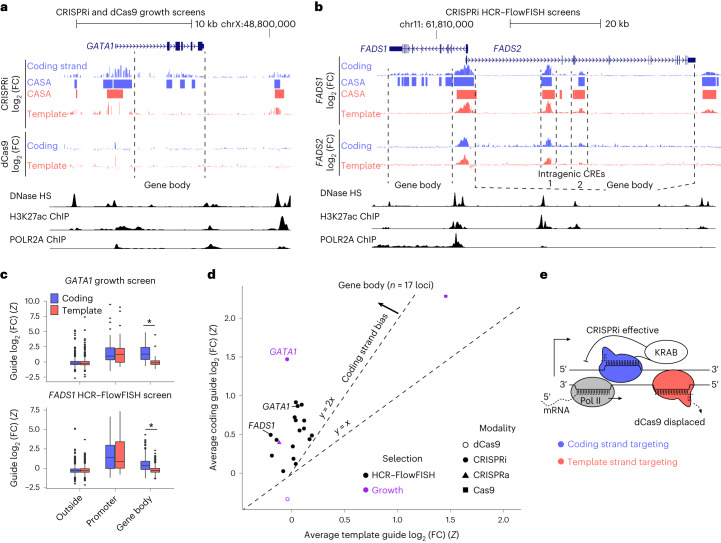
CRISPRi effects in the gene body are strand specific. **a**, Strand-specific CRISPRi growth screen affects tiling *GATA1*. CRISPRi and dCas9 tracks show the average of two biological replicates comparing day 21 to plasmid (*N* = 2,541 coding strand- and 2,263 template strand-targeting sgRNAs). **b**, Strand-specific CRISPRi HCR–FlowFish screen affects tiling *FADS1* and *FADS2*. CRISPRi tracks show the average of two biological replicates comparing high- and low-expression bins for the target gene (*n* = 4,609 and 4,942 sgRNAs per strand). **c**, Distributions of sgRNA effects (average of two biological screen replicates) in the gene body and at the promoter (within 2 kb upstream of the TSS), when sgRNAs are categorized by target strand in the (top) *GATA1* CRISPRi growth screen (*n* = 2,026, 1,731, 34, 27, 100 and 77 sgRNAs from left to right) and the (bottom) *FADS1* HCR–FlowFish screen (*n* = 3,121, 3,249, 90, 69, 520 and 702 sgRNAs). Boxes show the quartiles with a line at the median, vertical lines extend to 1.5 times the interquartile range, and dots show outliers. Asterisks denote significance with *P* < 1 × 10^–15^ by two-sided *t*-test. **d**, Strand specificity across screens tiling 17 loci for sgRNAs targeting the gene body. Each point is the average effect of all sgRNAs from a screen targeting that region averaged across two screen biological replicates, with color indicating the phenotypic readout and shape indicating the type of CRISPR perturbation. **e**, Proposed model of gene body strand bias.

**Extended Data Fig. 10:**
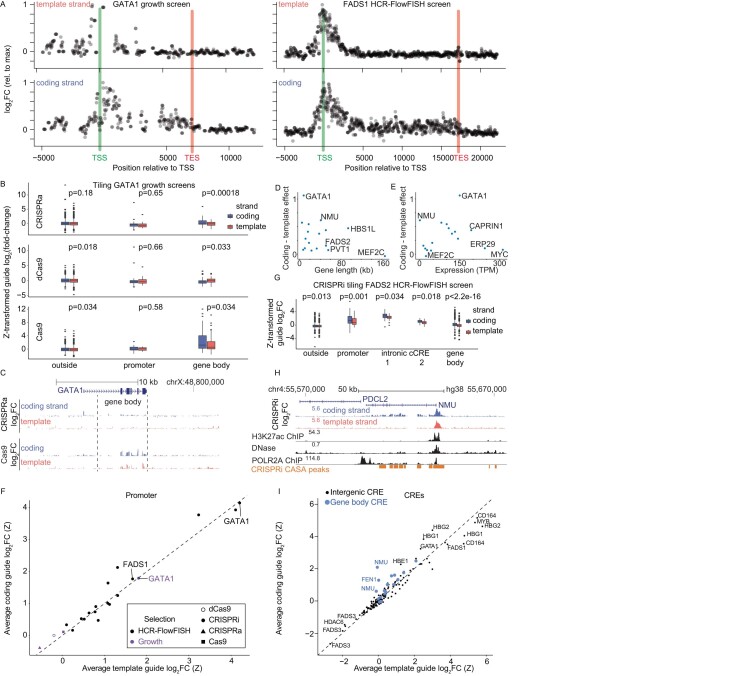
CRISPRi strand bias in the gene body. **A**) CRISPRi effects shown relative to the position of the TSS and transcription end site (TES). The TES is defined as the end of the transcript in UCSC RefGene (hg38). Points show average normalized sgRNA effect (n = 2 replicates). **B**) sgRNA effects in growth tiling screens using other modalities (CRISPRa, dCas9, or Cas9). Promoter refers to sgRNAs that are between the TSS and 2000 bp upstream of the TSS. Outside defined as outside the gene body, promoter, and K562 DHS peaks. P values show T-test for the comparison across strands. Boxes show the quartiles, with a line at the median, lines extend to 1.5 times the interquartile range, and dots show outliers (left to right: n = 2027, 1731, 35, 28, 101, 77 sgRNAs). **C**) CRISPRa and Cas9 tracks show the average of two biological replicates, comparing Day 21 to plasmid. **D**) Gene length compared with strand bias, defined as the difference between the median effect of coding strand-targeting and median of template strand-targeting sgRNAs. sgRNAs between the TES and 2000 bp downstream of the TSS are included, and genes less than 2000 bp are excluded (n = 17 loci with 2 replicates each). **E**) Strand bias similarly compared with expression level from RNA-seq in K562 cells (n = 20 loci). **F**) Points show the average effect of all sgRNAs targeting the promoter (n = 19 promoters with 2 replicates). **G**) sgRNA effects in a CRISPRi FlowFISH tiling screen for FADS2 regulatory elements. The two intronic CREs are defined as 500 bp windows centered on CASA peak calls and are annotated in Fig. 6b (left to right: n = 2105, 1935, 107, 126, 32, 26, 27, 19, 1940, and 1786 sgRNAs). **H**) Strand bias at a CRE within the gene body in a CRISPRi tiling HCR-FlowFISH screen of the *NMU* locus. **I**) Points show average effects of all sgRNAs targeting a CRE, defined as a 500 bp region centered on a K562 DHS that overlaps a CASA peak and is outside of the promoter (n = 2 replicates). CREs with ≥5 sgRNAs are included. Strand is defined with respect to the target gene (which may not correspond with transcriptional status of intergenic regions).

Most CRISPR screens model and analyze sgRNA effects without considering the potential impact of which DNA strand is targeted. Analyzing a CRISPRi growth screen tiling *GATA1*, we surprisingly found that sgRNAs targeting the coding strand affected growth, whereas template-targeting sgRNAs did not (*P* < 1 × 10^–15^; Fig. 6a). This difference was only observed in the *GATA1* gene body, perhaps related to RNA Pol II binding the template strand during gene transcription. We again observed significantly greater effects for sgRNAs targeting the coding strand within the gene body in the *FADS1* and *FADS2* HCR–FlowFISH CRISPRi tiling screens (*P* < 1 × 10^–15^; Fig. 6b,c). These coding strand effects were uniform throughout the transcribed gene body and ended at the transcription end site (TES; Extended Data Fig. 10a). We observed much weaker effects from the same library of sgRNAs targeting either strand in the gene body when using dCas9 alone (Fig. 6a) or when using CRISPRa (Fig. 6d and Extended Data Fig. 10b,c), suggesting that this phenomenon depends on the KRAB repressor (Fig. 6e). We propose a model wherein dCas9 binding could be reduced on the template strand due to competition with Pol II-mediated transcription, rendering KRAB ineffective. By contrast, when targeting the coding strand, KRAB can be effective.

To determine if this effect was present more generally, we expanded our comparison to 17 additional experiments (Methods). In all 17 CRISPRi screens, the average effect sizes of sgRNAs targeting coding strands within gene bodies were more than twofold higher than those targeting the template strands (Fig. 6d). The overall strand bias was not strongly associated with gene length or expression level measured by RNA sequencing (Extended Data Fig. 10d,e). In contrast to this strand bias in the gene body, there was no difference between coding and template strand sgRNA effects for all 17 corresponding promoters (Extended Data Fig. 10f).

Many enhancers reside within gene bodies^55^, motivating us to consider if these CRISPRi effects throughout gene bodies could be distinguished from effects at intragenic enhancers. *FADS2* contains intragenic enhancers, as determined by concordant signals from CRISPRi HCR–FlowFISH, DHS and H3K27ac ChIP–seq (Fig. 6b). In contrast to elsewhere in the gene body (and more similarly to intergenic enhancers), sgRNAs targeting both strands in these two enhancers had a significant effect on *FADS2* expression, although sgRNAs targeting the coding strand had a moderately greater effect than those targeting the template strand (*P* = 0.034 and 0.018, respectively; Fig. 6b and Extended Data Fig. 10g). This coding strand bias was present at some, but not all, intragenic CREs (for example, *NMU* and *CAPRIN1*; Extended Data Fig. 10h,i). These results demonstrate the necessity of considering strand to reliably identify intragenic CREs with CRISPRi.

## Discussion

CRISPR-based methods to examine CREs are an imperative step toward understanding the mechanisms that govern gene regulation and how disruption of these CREs contribute to disease. However, there are no common controls nor consensus on experimental design parameters, execution and analysis methods. This lack of a systematic comparison of screen sensitivity and specificity made evidenced-based sgRNA library design difficult, especially for modest-effect-size CREs or single-cell ‘omics readouts^56^. To address these limitations, we performed a comprehensive analysis of the ENCODE noncoding CRISPR screen datasets and proposed guidelines for screen implementation, standardized file formats and processed data expectations.

Our finding that the strongest enhancer-perturbing CRISPRi sgRNAs are nearest to distal CRE DHS summits is an important design criteria, potentially explained by accessibility improving CRISPRi efficiency, higher transcription factor motif density and/or more optimal sgRNA target sequences. Transcription-based screens are less susceptible to off-target effects than growth screens, potentially due to off-target sites impacting cellular proliferation more often than a single measured gene^17,47^. We report a CRISPRi strand bias specific to gene bodies that is particularly evident in non-CRE regions of gene bodies, similar to previous findings with Cas9 nuclease^57^. Whereas template strand-targeting sgRNAs with Cas9 show improvements for genome editing, our results suggest that CRISPRi is stronger with coding strand-targeting sgRNAs in the gene body and a need for strand-aware analysis to distinguish intragenic CREs from the subtle effects of CRISPRi throughout the gene body. After CRISPRi targeting, deposition of repressive H3K9me3 and diminished accessibility have been observed at the target CRE^18,25^, but such characterization is lacking for the vast majority of known CRISPRi-sensitive CREs.

We compared several peak callers for de novo CRE discovery in tiling screens and found that, although all identify positive-control CREs, CASA maintained both sensitivity and precision with fewer false positives from off-target noise. In sparse cCRE-targeting and cCRE/locus-tiling screens, including biological replicates and increasing sgRNA number were critical for detecting weak elements and improving power. We advise considering the thresholds described in this study for experimental coverage and sgRNA numbers as minimums and empirically evaluating power in other experimental systems, including single-cell ‘omics readouts that may suffer from data sparsity^58^. Likewise, we expect that future analytical packages will incorporate replication, strand bias and sgRNA efficacy to improve CRE detection.

An important limitation is that these experiments covered only 16 biosamples, with a strong emphasis on K562 cells due to data availability. Although we did validate key findings in mouse primary regulatory T cells, more systematic screening across phenotypes, cell types and genomic regions is needed to capture the range of *cis*-regulatory mechanisms. Guidelines for orthogonal CRISPR modalities (for example, CRISPRa) may differ from CRISPRi (as they differ at promoters^48^) and may be biased by library designs, phenotypic readouts, specific genomic loci perturbed and analysis methods used in these experiments. Building a larger, more diverse collection of CREs will improve guidelines for selecting sgRNAs and will empower refinement and benchmarking of methodological guidelines and analysis techniques. Although others have found limited evidence for regulatory function outside known K562 cell DHSs or H3K27ac sites^59^, previous studies have also identified putative repressor elements via CRISPRi perturbations, including a REST-driven repressor of FADS3 (ref. ^9^) as well as evidence of silencer elements using reporter assays^60,61^.

Optimal experimental and analytical parameters are needed to increase the scale and/or sensitivity of CRISPR screens, especially as they are increasingly applied with multiplexed readouts and in single-cell schemas^8,59^. Recommendations based on bulk CRISPR screens, such as prioritizing sgRNAs targeting the DHS peak, should apply to single-cell screens, but minimum sgRNA number per cCRE and optimal cell and/or sequencing coverages will likely differ. Currently, the most extensive published single-cell dataset uses two sgRNAs per target, precluding an in-depth analysis of optimal sgRNA density per cCRE^44^. Based on a diverse set of CRISPR screens in the ENCODE database, along with predesigned sgRNAs for cCREs, this work will accelerate the functional characterization of regulatory elements across the genome and make noncoding CRISPR screening methods accessible to the broader community.

## Methods

### Cell lines and cell culture

K562 cells with a doxycycline-inducible CRISPRi blue fluorescent protein (BFP) were a gift from the Lander lab (Broad Institute, Cambridge, MA, USA) and were identical to those used in a previous study^9^. In that study, the cells were generated by (1) transducing K562 cells with a construct expressing reverse tetracycline transactivator linked by IRES to a neomycin resistance cassette expressed from an *EF1α* promoter (ClonTech) and selecting with 200 µg ml^–1^ G418 (Thermo Fisher) and (2) transducing these reverse tetracycline transactivator-expressing K562 cells with a KRAB-dCas9 construct. Cells expressing BFP were selected by fluorescence-activated cell sorting. Cells were grown in RPMI-1640 GlutaMAX (Gibco) with 10% heat-inactivated fetal bovine serum (Gibco).

### *GATA1* screen with varied cell coverage

A previously described noncoding *GATA1* lentiviral library was used^9^. CRISPRi BFP was induced for 24 h with a final concentration of 1 μg ml^–1^ doxycycline (VWR). Active CRISPRi was checked by confirming that doxycycline-induced BFP signal was observed in >90% of cells by flow cytometry (Sony, MA900). Cells were grown for 2 weeks after transfection, following the HCR–FlowFISH protocol exactly as previously described^9^. High- and low-expression bins (top and bottom, 10% each) were also gated following the previous HCR–FlowFISH protocol^9^. Cells were sorted at multiple folds of library size (25×, 50×, 100× and 200×).

### The ENCODE CRISPR Screen Database and overlap with cCREs

Individual sgRNAs were aggregated across fully released experiments with sgRNA-level and/or element-level quantification files performed in human cell lines using the November 2022 data release excluding single-cell gene expression readouts (Supplementary Table 1; ‘included_in_all_meta’, *n* = 75). Note that three experiments were removed in the August 2022 data release. These experiments have been rereleased as of November 2022 but were excluded from all calculations. The coordinates of each sgRNA were adjusted based on the type of perturbation used in the corresponding experiment (Cas9 cutting: ±10 bp of PAM, dCas9-KRAB: ±150 bp of PAM) and lifted from hg19 to hg38 genome builds when necessary. For 15 sgRNAs that did not have strand information in the associated elementReference or guideQuant files, the protospacer sequences were manually aligned to the hg19 genome build to retrieve the strand information before adjusting for the perturbation modality. For paired sgRNA experiments, we considered each gRNA in a given pair as a unique perturbation and adjusted the coordinates as described above. The total number of perturbations was defined as the number of unique coordinate combinations after adjusting for the perturbation modality. These perturbation regions were then intersected (bedtools intersect) with 100-bp tiled bins across each chromosome, followed by merging of overlapping bins (bedtools merge -d 1), and the percentage of the human genome perturbed was calculated by dividing the sum of bases within the tiled bins by the effective genome size (3,088,269,832 bp). The significant CREs from each experiment (defined by the contributing lab) were intersected with the same 100-bp tiled bins and similarly merged to generate the final CRE set (Supplementary Table 2).

#### K562 cell screen integrated analysis

Individual sgRNAs were aggregated across released experiments performed in K562 cells with FlowFISH-based readouts with sgRNA-level and/or element-level quantification files (November 2022 data release, excluding single-cell gene expression readouts; Supplementary Table 1, ‘included_in_k562_meta’). The coordinates of each sgRNA were adjusted based on the type of perturbation used in the corresponding experiment as described above and were lifted from hg19 to hg38 genome builds when necessary. These perturbation regions and the CREs from each experiment (defined by the contributing lab) were then intersected with 100-bp tiled bins as described above to generate the perturbed and CRE sets, respectively. The CRE coordinates and feature overlap are provided in Supplementary Table 5.

The genomic and epigenomic annotation files used for enrichment testing and signal comparison are provided in Supplementary Table 4. The perturbed regions and CREs were intersected with the significant peak calls or predicted ENCODE SCREEN cCREs (‘features’). A two-sided Fisher’s exact test was performed comparing the number of features overlapping a CRE to the total number of features perturbed. The results are reported in Supplementary Table 6. The UpSet plot comparing CRE overlap with features was generated using the R package ‘UpSetR’. To compare the signal of each feature between perturbed regions and CREs, bigWig files were converted to bedgraph format using the University of California Santa Cruz utility ‘bigWigToBedGraph’. Next, the perturbed regions and CREs were intersected with the bedgraph files containing FC over background signal (‘signal’). Signal values were then normalized by dividing by the element size, and a two-sided Wilcoxon test was performed comparing the median signal for each feature between perturbed, not significant regions and CREs. Two-sided Wilcoxon test and Student’s *t*-test results and median, mean and standard deviation of normalized signal values are reported in Supplementary Table 7.

#### CRE features in additional cell types

We retrieved the CREs (defined by the contributing lab) from the ‘elementQuantification’ files for each experiment and lifted hg19 to hg38 coordinates when necessary. The sources for the peak calls for each ‘feature’ are listed in Supplementary Table 18. The CREs were intersected with peak calls corresponding to a given feature. For WTC11 iPSCs, the UpSet plot comparing the CRE overlap to accessible chromatin regions and histone mark ChIP–seq was generated using the R package UpSetR. The count and proportion of CREs overlapping each feature in all ten cell lines analyzed are reported in Supplementary Table 8.

#### CRISPR screen comparisons with individual sgRNA validations

sgRNA abundance and element activity values from CRISPR screens and results from experimental validations were obtained from supplemental materials from each of the cited publications. Two-sided Pearson correlation values and associated *P* values between the validation assays and screen results were calculated using the ‘stat_cor’ function from the R package ‘ggpubr’.

#### Cross-screen analysis at *GATA1* and *MYC*

hg38 PAM coordinates were used to uniformly analyze and compare the five CRISPR screens from various labs. For screens with hg19 coordinates, their protospacer coordinates were first mapped to hg38 using bowtie1 and the ‘-n–best’ options. The hg38 PAM coordinates for each screen were then extracted by taking the 3 bp downstream of each protospacer, which were confirmed to contain the expected NGG sequence. For the *GATA1* locus, 250 such PAM coordinates were found to be shared across the five screens, and these common PAM coordinates were filtered out for their sgRNA GuideScan target specificity (>0.2), leading to 176 PAM coordinates that were used for pairwise effect size comparison of the five screens. Effect sizes were computed using mean-normalized log_2_ (FC) (Eq. 1 provided in Cell coverage/sorting depth titration experiments for HCR–FlowFISH). To compare the effects of CRISPR–Cas9 and CRISPRi at exons and DHSs, we obtained subsets of sgRNAs with significantly high log_2_ (FC) effect sizes (*Z*-score *P* < 0.001). We then extracted significant sgRNAs that target exons or K562 cell DHSs by overlapping their PAM coordinates with Ensembl-annotated exons and K562 cell DHSs obtained by extending K562 cell DHS narrow peaks (ENCFF899KXH) by 500 bp in both directions from their centers. For CRE annotations in the Cas9 versus CRISPRi comparison of effect sizes, sgRNAs were defined as targeting eGATA1 if their start position was within 48641136 and 48641797, eHDAC6 if their start position was within 48658755 and 48659455 or *GATA1* TSS if their start position was within 48644481 and 48645481.

#### ABC model CRE target predictions

We downloaded the ABC predictions for K562 cells^62^ and evaluated the percentage of significant CREs identified in the HCR–FlowFISH screens that regulate the target gene predicted by ABC. ABC-predicted CRE–gene links were based on average HiC using an ABC score threshold of 0.015 for significant predicted links. CREs from the screens were intersected with the cCRE ranges provided by the K562 cell ABC predictions without any additional coordinate expansions.

#### Evaluating sgRNA effects in DHS or H3K27ac peaks

Significant, non-TSS-overlapping distal enhancer elements identified in any of the HCR–FlowFish screens that intersect both a DHS and H3K27ac peak were first selected. For each enhancer element, we calculated the mean effect of all sgRNAs within its intersecting DHS or H3K27ac peak region. The sgRNA intersections used the sgRNA’s 3-base PAM coordinate window.

#### Evaluating sgRNA effects as a function of distance from the DHS summit

Significant, non-TSS-overlapping distal enhancer elements identified in any of the HCR–FlowFish screens that intersect both a DHS and H3K27ac peak were selected. We then selected all sgRNAs within 2 kb of the enhancer element’s strongest intersecting DHS summit and normalized their effect sizes to the mean of all sgRNAs intersecting that DHS peak (using the sgRNA’s 3-base PAM coordinate window).

To produce plots of DNase-seq, H3K27ac ChIP–seq and normalized sgRNA effects relative to the DHS peaks, we took the sgRNA coordinates around significant, nonpromoter enhancers and expanded them each by ±150 bp to conservatively approximate KRAB’s repressive window and assigned each base position that sgRNA’s normalized effect size. If multiple expanded sgRNA windows overlap, then their effects were averaged per base position. These data were converted into a bigWig file, and we used deepTools to plot the distance-dependent sgRNA effects along with DNase-seq and H3K27ac ChIP–seq signal tracks. Because of the noise present in the GITR screen, only significant, nonpromoter enhancers with an effect size of ≤–1 were included in the sgRNA effect analyses.

#### Evaluating significant CREs as a function of location within the same TAD as their target gene

Significant CREs in K562 cell screens with adjusted *P* values of ≤0.05 that reside inside a K562 cell HiC TAD (ENCFF173VDJ) were included for analysis. Sixty-five significant CREs were not in a TAD and were excluded. For each CRE’s target gene, it was determined if the consensus RefSeq promoter 1-kb window around the TSS was in the same TAD as the CRE.

#### Effect size-dependent sgRNA number per element power analysis

For the guide downsampling analysis, we took guide-level effect sizes from the CRISPRi FlowFISH screens targeting the *GATA1* locus and averaged the effect sizes from two biological replicates. We then took the sgRNAs targeting the eGATA1 enhancer and rescaled their effects so that the average of all 37 sgRNAs was a 0–50% perturbation, in steps of 10%, of *GATA1* expression. For each number *n* of sgRNAs, we sampled *n* sgRNAs from the scaled distribution, computed a Welch’s *t*-test *P* value (equal_var = False, dof = 1) against all nontargeting negative-control sgRNAs, performed a Benjamini–Hochberg correction with all elements tested in the screen and tested for false discovery rate (FDR) < 0.05. We repeated this procedure 500 times for each (effect size, guide number) pair and computed power as the fraction of times we correctly rejected the null hypothesis.

#### Off-target sgRNA enrichment analysis

For each respective screen, we selected sgRNAs located at least 1 kb away from any DHS peak, regardless of significance, or significant element. We used GuideScan to obtain sgRNA aggregated CFD scores, a summary score of off-target specificity based on the weighted likelihood of off-target activity across a full list of potential off-target sites and separated sgRNAs into low specificity (CFD < 0.2) or high specificity (CFD ≥ 0.2). We then calculated the proportion of sgRNAs in each specificity category that had effect sizes more than two times the standard deviation of negative controls from the mean of the negative controls and performed a Fisher’s exact test to derive a *P* value for each OR.

#### Safe versus nontargeting negative-control variance statistical analysis

For Extended Data Fig. 8, negative-control sgRNAs were subsampled 1,000 times each in increasing increments of ten sgRNAs. For each subsample, we performed a Levene’s test against the full set of 1,000 of the respective type of negative-control sgRNAs. We then calculated the percentage of times that the result of the Levene’s test was significant (*P* < 0.05; that is, the number of times variance between the subset and the whole set was statistically different) from the 1,000 subsamples for each increment. This percentage is the empirical *P* value, such that the black threshold line of *P* = 0.05 means that out of 1,000 subsamples, only 50 had significantly different variances compared to the variance of the full set of that respective type of negative-control sgRNA.

#### Promoter-targeting ‘positive-control’ sgRNA selection analysis

For Extended Data Fig. 8c, we selected all TSSs provided by the FANTOM5 database that passed a relaxed Timo TSS classification score of 0.14 for the genes measured by HCR–FlowFISH. We calculated the average effects of the ten closest sgRNAs to each TSS position. Where a TSS window was provided, we used the first transcribed base position to calculate absolute sgRNA distances. To compare these sgRNAs against those provided by genome-wide CRISPRi libraries (Broad Dolcetto^49^ and hCRISPRi-v2 (ref. ^48^)), we selected the sgRNAs whose spacers matched those tested in the HCR–FlowFISH screening libraries; the sgRNAs from hCRISPRi-v2 follow a G + 19 base spacer convention, so the 5′-most base from the HCR–FlowFISH spacer sequences was trimmed to facilitate spacer sequence matching. Because these libraries often provided lower scores than the optimal TSS, we aimed to provide a heuristic method of selecting TSS-targeting sgRNAs by selecting the TSS with the greatest Pol II ChIP–seq signal (TSS provided by RefGene, total Pol II ChIP–seq signal was calculated in a window ±500 bp around the TSS) and picking the ten nearest sgRNAs.

### Cell coverage/sorting depth titration experiments for HCR–FlowFISH

HCR–FlowFISH experiments at *GATA1* were performed using guide libraries, K562 cell lines, transcript detection, sorting and sequencing strategies, as previously described^9^, and following guidelines suggested here (Supplementary Information Section 7). To evaluate the effects of sampling cell numbers at different levels of complexity, defined as the number of observations per number of sgRNAs used, we performed two replicates of the *GATA1* library and partitioned them into different sorting depths. The same library was sorted into 20×, 50×, 100× and 200× the guide library size. To assess the impact of sequencing complexity, each sorting strategy was sequenced at a depth of more than 2,000×.

Effect size of each sgRNA was computed using Eq. 1 to underweight sgRNAs with low read counts by normalizing read counts by their mean:

1 $$\begin{array}{l}{\rm{Mean}}-{\rm{normalized}}\,\log_2{({\mathrm{FC}})_i}\\=\log_2\left(\left(1+[{A_i}/{{\mathrm{mean}}}(A)]\right)/(1+[{B_i}/{{\mathrm{mean}}}(B)])\right)\end{array}$$

2 $$\begin{array}{l}{\rm{Linear}}-{\rm{transformed}}\,\log_2{({\mathrm{FC}})_i}\\=\log_2\left([(1+{A_i})/{{\mathrm{sum}}}(A)]/[(1+{B_i})/{{\mathrm{sum}}}(B)]\right)\end{array}$$

where *A* and *B* are each vectors encoding the number of reads for each guide in low- and high-sort bins, respectively. Target coordinates for each sgRNA were determined by their target PAM coordinates. Coordinates for the *GATA1* CREs were obtained using HCR–FlowFISH CASA CRE annotation (ENCFF413WYU).

### Bootstrap sampling analysis for simulating CRISPR screens performed at various sequencing depths

Bootstrap sampling analysis for sequencing depth was performed using ENCODE standard guide quantification files, which record the number of sequencing reads that map to each sgRNA sequence in a given library. Each CRISPR screen comes with two guide quantification files. For sorting-based screen approaches (for example, FlowFISH), one file quantifies the number of mapped sequencing reads in low-expression sorted bins (labeled ‘A’), whereas the other file quantifies those in high-expression sorted bins (labeled ‘B’). For growth-based screen approaches, we quantify using samples collected from an earlier time point (‘A’) and a later time point (‘B’). To simulate an experiment with sequencing depth of *d*, we sampled with replacement total *N*  × *d* number of reads independently from each A and B, where *N* is the number of distinct sgRNAs*N*in×*d*a simulate an experiment with sequencing depth *d*, we sampled with replacement total *N* × *d* number of reads independently from each A and B, where *N* is the number of distinct sgRNAs in a library.

For the CRISPR screens used for the bioreplicate reproducibility and dropout analyses, reads were sampled independently for each of the two bioreplicates (A1, A2, B1 and B2). sgRNAs that had 0 mapped reads in any one of A1, B1, A2 and B2 were excluded from the analyses. At each value of *d*, 100 independent bootstrap samples were generated to be used for dropout and bioreplicate reproducibility analyses (Fig. 3f,g).

For the dropout simulation analysis, we defined dropout sgRNAs as those that resulted in less than ten sampled reads from either A_sampled_ or B_sampled_. For bioreplicate reproducibility analysis, we computed Pearson correlations of log (FC) effect sizes (log_2_ [(1 + A_sampled_)/(1 + B_sampled_)]) from every pair of bootstrap samples, one coming from bioreplicate 1 and the other coming from bioreplicate 2.

### Peak caller comparisons

#### aggrDESeq2

For each experiment, read counts of individual sgRNAs for the initial and final time points were obtained from the guideQuant files. Differential abundance testing was performed using the DESeq2 package with default parameters, with contrasts defined such that the average log_2_ (FC) values of sgRNAs more abundant in the final time point or high-expressing bin have positive values. Next, 100-bp bins were tiled across chromosomes containing perturbations. Coordinates for individual sgRNAs were adjusted based on the perturbation modality (Cas9 cutting: ±10 bp of PAM; dCas9: ±10 bp of PAM; dCas9-KRAB: ±150 bp of PAM) and intersected with the bins. For every 100-bp bin, a significance value was calculated using Fisher’s method for aggregating *P* values with the unadjusted DESeq2 *P* values as input. The aggregated *P* values were then FDR adjusted. Significant bins were defined as FDR < 0.01. Note that sgRNAs that intersect more than one bin contribute to the calculations for all overlapping bins. This was repeated without filtering out sgRNAs with GuideScan specificity scores of <0.2. To determine if the method was sensitive to outliers, we removed the most significant sgRNA per bin and recalculated the bin significance and effect size. For the *Gitr* locus screen, the above process was repeated.

#### CASA

sgRNA guideQuant files were parsed to provide genomic mapping coordinates of the protospacer sequence and raw guide counts per experimental condition in the CASA input format. We ran a containerized deployment (https://hub.docker.com/r/sjgosai/casa-kit; version 0.2.3) on the Google Cloud Platform using a wrapper script provided in the CASA GitHub repository (https://github.com/sjgosai/casa). CASA was run using a sliding window of 100 bp in width and step size and a ROPE threshold of 0.693 (that is, the default settings). As in previous work^9^, peaks that were supported by at least ten sgRNAs and were shared between two bioreplicates were reported.

#### CRISPR-SURF

sgRNA guideQuant files were parsed according to the input format required for CRISPR-SURF (in particular, converting PAM coordinates to protospacer coordinates). SURF_count was then run with the options -nuclease cas9 -pert crispri to produce an input file for deconvolution. SURF_deconvolution was run using the -pert crispri option, and the resulting negative_significant_regions.bed was used to identify positive regulators of expression with FDR < 0.05. CRISPR_SURF was run using the provided Docker container using Singularity.

#### MAGeCK

sgRNA guideQuant files and coordinate expansion were performed similar to as described above. One hundred-base pair bins were created by taking the first most upstream coordinate position among all sgRNAs in the respective screening library and creating 100-bp bins until reaching the most downstream sgRNA coordinate position. Expanded coordinate sgRNAs were then intersected with the bins. MAGeCK was run using the default parameters (–norm-method = median –sort-criteria = negative –remove-zero = none –gene-lfc-method = median), and only the significance values corresponding to the expected effect size direction for each screen (negative for the growth screens and positive for the FlowFISH screens) were used to calculate significance, which was calculated similar to as described above.

#### RELICS

sgRNA guideQuant files were prepared to provide genomic coordinates and raw counts of each sgRNA in the standard input format for RELICS. The sgRNAs overlapping promoter regions and exons of each target gene were labeled as functional sequences for CRISPRi screens and CRISPR–Cas9 screens, respectively. CRISPR systems used for each screen were specified for RELICS. The functional sequences were then identified for each screen using the default settings for RELICS v.2.0 (min_FS_nr:30, glmm_negativeTraining:negative_control).

#### Pairwise Jaccard similarity

For each method, peaks were loaded, and a set was constructed with all nucleotides in the tiled region called significant. For each pair of peak calling methods, the Jaccard similarity was computed as

$$\frac{{\rm{|}}A\bigcap B{\rm{|}}}{{\rm{|}}{\rm{A}}\bigcup {\rm{B}}{\rm{|}}}$$

For the ‘Canonical Elements’, we used the coordinates of the *GATA1* promoter (hg38 chromosome X: 48786330–48786733), eGATA1 (chromosome X: 48782816–48783227) and eHDAC6 (chromosome X: 48800584–48800859).

#### Effect sizes within peaks

For comparison of the distribution of guide effects (log_2_ (FC)) for the sgRNAs falling within peaks identified by different peak callers, we started by using Eq. 2 to calculate the log_2_ (FC) for each guide. We then picked the sgRNAs that overlapped with the called peaks for each analysis tool and plotted the log_2_ (FC) values of the filtered sgRNAs.

#### Nucleotide overlap with annotations

Peaks identified by different CRISPR cCRE callers were intersected with ENCODE (DHS: ENCSR000EKS; H3K27ac: ENCSR000AKP) and SCREEN annotations (Supplementary Table 18).

#### Intersection of CRE calls

Significant CRE calls from each peak caller were intersected using bedtools multiinter. The output was used to generate the UpSet plots using the ‘upset’ function within the R package UpSetR.

### Comparison of time points

A CRISPRi growth screen with sgRNAs tiling the *GATA1* locus (ENCSR719QWB) was used to analyze the effect of time point selection. CASA peak calls were generated as described above. Relatedly, a CRISPRi HCR–FlowFISH screen at the *GATA1* locus (ENCSR917XEU) was inspected for dropout due to potential growth effects.

### Strand-specific quantification of sgRNA effect sizes

All CRISPR screens used in this analysis had specific gene targets (CRISPRi growth screen tiling across the *GATA1* locus and HCR–FlowFISH), and their sgRNAs were unambiguously labeled as either template strand- or coding strand-targeting sgRNAs depending on which strand their protospacers were located relative to the transcriptional directions of their target genes (Fig. 6a,b). For the *GATA1* CRISPRi growth screen, sgRNAs were filtered for GuideScan aggregated CFD specificity scores of >0.2 to remove sgRNAs with off-target growth effects. We then labeled each sgRNA as gene targeting if its PAM sequence was located between 2,000 bp downstream of TSS and TES. The 2,000 spacers were used to exclude gene body-targeting sgRNAs that were TSS proximal and affected promoter activities. sgRNAs with PAM sequences located between 2,000 bp upstream of the TSS and the TSS itself were labeled promoter targeting, and all other sgRNAs were labeled ‘outside’ (Fig. 6c). RefGene annotations were used to identify TSSs and TESs for each gene, and for genes with multiple isoforms, isoforms with the highest levels of K562 Pol II ChIP–seq signals (ENCFF914WIS, signal *P* values) at both the TSS and TES were used. Based on the results of the HCR–FlowFISH screen, it appeared that *PVT1* was primarily expressed from an alternative TSS in K562 cells. This position overlaps the CRE termed e3 in a previous K562 screen^10^ (but was not included as a TSS in RefGene), and we used its position (chromosome 8: 128045692) as the TSS of the *PVT1* gene for length analyses. Three of 20 HCR–FlowFISH experiments were excluded from this analysis (Fig. 6d), as they had less than five tested protospacers located within template strand promoters, coding strand promoters, template strand gene bodies or coding strand gene bodies.

### Chromatin accessibility measurement in primary mouse regulatory T cells

Chromatin accessibility was measured using the Omni-ATAC protocol^63^ on 50,000 sort-purified CD4^+^Foxp3–GFP^+^ regulatory T cells that had been differentiated in vitro from sort-purified naive CD4^+^ T cells from C57BL/6 mice.

### Stain-and-sort screen for *Gitr* expression in primary mouse regulatory T cells

Twelve ATAC-seq peaks within 50 kb of the *Gitr* (*Tnfrsf18*) locus in regulatory T cells were selected for gRNA design using GuideScan2. The resulting gRNAs were filtered to keep those with a specificity score of ≥0.2, to remove repeats of GGGGG and TTTTT and to restrict guides that overlap by more than 5 bp. This left 404 targeting sgRNAs to which 40 nontargeting gRNAs were added as negative controls.

The gRNA library was cloned into a mouse stem cell virus retroviral mU6 promoter-driven expression system using NEBuilder HiFi DNA Assembly (New England Biolabs, E2621L). This retrovirus contains a *Thy1* reporter gene under the control of a separate *Pgk* promoter. gRNA containing retrovirus was produced using the Platinum-E Retroviral Packaging Cell Line (Cell Bio Labs, RV-101) following transient transfection.

Naive CD4^+^ T cells were then collected from the spleen and lymph nodes of Foxp3–eGFP dCas9-KRAB CD4-CRE C576BL/6 mice using magnetic selection (Thermo, 8804-6821-74)^64^. Four mice were used as independent biological replicates. Cells were seeded at 0.5 × 10^6^ cells per ml and cultured in complete RPMI (10% fetal bovine serum, 1% penicillin, 1% streptomycin, 1% gentamicin, 1% l-glutamine, 1% HEPES, 1% sodium pyruvate and 55 nM 2-mercaptoethanol) and activated under Th0 conditions (250 ng ml^–1^ anti-CD3, 1 µg ml^–1^ anti-CD28, 2 µg ml^–1^ anti-interleukin-4 (IL-4) and 2 µg ml^–1^ anti-interferon-γ). Cells were transduced at 24 h with viral supernatant containing 6.66 ng µl^–1^ polybrene and at 900*g* for 2 h at 30 °C. Cells were then cultured under regulatory T cell polarizing conditions (Th0 conditions + 10 ng ml^–1^ IL-2 and 10 ng ml^–1^ human transforming growth factor-β) for 96 h. Live cells were stained for viability with e780 (Thermo, 65-0865-14), GITR-PE (BD Bioscience, 558140), CD4-e450 (Thermo, 48-0042-80) and THY1.1-APC (Stem Cell Technologies, 60024AZ) for 30 min on ice and sorted using a Sony SH800Z with a 70-µm chip. At least 40,000 cells were sorted from the top and bottom 15% of GITR signal (gating: lymphocytes/live/singlets/CD4^+^/THY1.1^+^/Foxp3–eGFP^+^/GITR^hi/lo^). gDNA was recovered using a Zymo Quick-DNA Miniprep Plus kit (Zymo, D4068), and gRNA was recovered via PCR. Libraries were sequenced on an Illumina MiSeq using 20-bp single-end reads.

### Reporting summary

Further information on research design is available in the Nature Portfolio Reporting Summary linked to this article.

## Online content

Any methods, additional references, Nature Portfolio reporting summaries, source data, extended data, supplementary information, acknowledgements, peer review information; details of author contributions and competing interests; and statements of data and code availability are available at 10.1038/s41592-024-02216-7.

### Supplementary information

Supplementary Information Supplementary Figs. 1–11 and Discussion.

Reporting Summary

Supplementary Tables 1–11 and 13–18 Supplementary Tables 1–11 and 13–18.

Supplementary Table 12 sgRNA library and read counts for *GATA1* titration experiments.

## Data Availability

The genomic and epigenomic annotation files used in this analysis are provided in Supplementary Table 4. Accession IDs for public datasets used in this study are provided in Supplementary Table 18. All CRISPR screen datasets used in this study are available in the online ENCODE portal, and accession IDs are included in Supplementary Table 1. sgRNA counts for the *GATA1* titration experiments are provided in Supplementary Table 11. The *Gitr* regulatory T cell screening data can be found at https://www.dropbox.com/scl/fo/7q92wt7zyejfkwetsgsr6/h?rlkey=30ytwfaazty33bz3ez30coiy8&dl=0. Public CSC track hub repositories to visualize CRISPR screen data and results are available for Figs. 1 (https://data.cyverse.org/dav-anon/iplant/home/joh27/track_hub_fig1/hub.txt) and 6 (https://data.cyverse.org/dav-anon/iplant/home/ohjinwoo94/track_hub_fig6/hub.txt).

## Code availability

The code for CASA can be found at https://github.com/sjgosai/casa. The code for using GuideScan2 to design sgRNAs for all cCREs can be found at https://github.com/schmidt73/encode_pipeline. GuideScan2 is available with a web interface at https://guidescan.com/. The code used for other analyses is available online at https://github.com/Reilly-Lab-Yale/ENCODE-CRISPR.
