# Supplementary material for: Multicenter integrated analysis of noncoding CRISPRi screens
Source: Nat Methods. 2024 Mar 19;21(4):723–34. doi: 10.1038/s41592-024-02216-7 (PMC11009116; doi:10.1038/s41592-024-02216-7)
Supplement: Supplementary file 1 — Supplementary Figs. 1–11 and Discussion. [file 41592_2024_2216_MOESM1_ESM.pdf]

nature methods

Analysis

https://doi.org/10.1038/s41592-024-02216-7

Multicenter integrated analysis of noncoding CRISPRi screens

In the format provided by the authors and unedited

Supplementary Material Related to:

Multi-center integrated analysis of non-coding CRISPRi screens

Table of Contents

Supplementary Sections **2**

Supplementary Section 1. sgRNA Sequence and Coordinate Mapping 2

Supplementary Section 2. Navigating CRISPR Screening Data on the ENCODE Portal 3

Supplementary Section 3. Validation of CREs identified from CRISPR screens 6

Supplementary Section 4. Design of sgRNA libraries targeting ENCODE SCREEN cCREs 7

Supplementary Section 5. Standardized non-coding CRISPR Screen File Formats 8

Supplementary Section 6. Processed non-coding CRISPR screen file formats 9

Supplementary Section 7. Expression-based non-coding CRISPR screening 10

Supplementary Figures **12**

Supplementary Fig. 1. Individual sgRNA validations correlate with non-coding CRISPR screen results 12

Supplementary Fig. 2. GuideScan2-designed sgRNAs targeting all cCREs from the ENCODE SCREEN portal. 13

Supplementary Fig. 3: Guide drop out rate with varying HCR-FlowFISH sorting depths 14

Supplementary Fig. 4: Effect size normalization methods affect biological replicate correlation 15

Supplementary Fig. 5. Overlap between peak calls on specificity-filtered CRISPRi tiling screen of GATA1 locus 16

Supplementary Fig. 6. Peak calls without filtering out low specificity sgRNAs 17

Supplementary Fig. 7. Mapping sgRNAs to reference genome for data standardization 18

Supplementary Fig. 8. ‘Functional Characterization’ card on the ENCODE home page 19

Supplementary Fig. 9. Filtering search results in the ENCODE portal 20

Supplementary Fig. 10. An experiment series summary page 21

Supplementary Fig. 11. The ‘Files Section’ of the experiment series summary page 22

Supplementary Tables **23**

Public datasets accessed 24

Author contributions **24**

Acknowledgements **24**

Conflict of Interest Statements **24**

Supplementary References 26

Supplementary Sections

Supplementary Section 1. sgRNA Sequence and Coordinate Mapping

As sgRNA design and synthesis strategies are variable, we found direct comparison of guide sequences poorly reflects commonalities between screens. Instead, guide sequences converted into their PAM coordinates in a common genome build (hg38) (**Methods**) resulted in improved overlap (percent overlap with sequence match vs PAM method for *MYC* and *GATA1*). We also compared the effects of lifting over PAM coordinates between genome builds vs. mapping guide sequences with short read aligners to harmonize screening data, and we observed that, for 3 out of 4 guide library constructed in hg19, bowtie had higher rate of unique and exact mapping of sgRNA to hg38 genome build (**Supplementary Fig. 7**).

Supplementary Section 2. Navigating CRISPR Screening Data on the ENCODE Portal

The ENCODE portal has been updated to support submission and subsequent navigation of functional characterization assay data allowing users to navigate these data along various facets: biosample, organism, readout and target, perturbation modality and scale of perturbations, and genome build. In total, the portal contains 119 CRISPR screen experiments, comprising 106 experiments performed in human cell lines and 13 experiments performed in mice as of August 2022 (**Supplementary Table 1**). The most recent data release (November 2022) increased the total number of experiments to 129 (116 in human cell lines, 13 in mice) and we anticipate the release of additional experiments by the end of 2022.

The navigation guidelines covered here allow rapid exploration and filtering of functional characterization datasets generated and made available by the ENCODE Consortium. It guides users through the ENCODE portal to search, visualize and download experiment series data using a web browser (**Supplementary Fig. 8-9**). The functional characterization datasets available in the portal were generated by assays which study the relationship between a DNA sequence and its regulatory activity, such as Massively Parallel Reporter Assays and CRISPR screens.

Find Functional Characterization datasets on the portal

1. Navigate to the ENCODE portal home page at https://www.encodeproject.org/.

2. Locate and click on the “Functional Characterization" card (**Supplementary Fig. 8**).

3. The search result page lists the available functional characterization data. As of August 2022, the ENCODE portal had 477 functional characterization datasets listed (note that only 25 are listed on the page by default)

4. Filter the search results :

    - The sidebar on the left side of the search results page is populated with facets that allow users to filter search results using different criteria. Locate the “Quality” facet group and click on it, notice that the “released” entry under the “Status” facet is highlighted in blue, indicating that the search results have been filtered for datasets that have been released to the public.

    - Selections can be applied to several facets at a time and the combined filters possess an “AND” relationship. Scroll back up to the facet group “Provenance” and under the facet “Lab” select “Pardis Sabeti, Broad”. Now the search results have been filtered to include only functional characterization datasets that have been performed in Dr. Sabeti’s laboratory AND have been released to the public.

    - Review the list of search results. As of August 2022, the facets selections above returned 20 series datasets. Select the dataset ENCSR408VHJ by clicking on its title “CRISPRi Flow-FISH screen in K562 with HCR-FlowFISH readout of MYB” (**Supplementary Fig. 9**).

Navigate Experiment Series Summary Page

The experiment series summary page is organized in six distinct sections: (A) Page Title, (B) Summary, (C) Attribution, (D) Experiments, (E) Control Experiments and (F) Files section (**Supplementary Fig. 10**).

- The “Summary” section (**Supplementary Fig. 10B**) contains key information about the series, including but not limited to donor, assay, biosample summary, diseases, treatments. It will also include a link to the elements reference dataset that contain information about the investigated functional elements or genomic loci.

- The “Attribution” section (**Supplementary Fig. 10C**) lists the lab, award and project. It will specify if there are aliases used for the series and if there are cross-references to the dataset in other public repositories (external resources).

- The “Experiments” section (**Supplementary Fig. 10D**) lists in a table all functional characterization experiments that have been included in the series. The first column of the table provides the accessions of the experiments, followed by the assay type of the experiment. For experiments that measure expression readout of a specific genomic locus, the column “Examined loci" will be populated with the relevant information.The next column includes biosample summary, followed by columns listing the lab and status information. Additional information about the various statuses of the experiments and other objects on the portal is available at https://www.encodeproject.org/help/getting-started/status-terms/. The cart option is the last column in the Experiments section table. It allows grouping of individual experiments using the cart mechanism. Note, that you need to create an ENCODE portal user account to take advantage of the cart features. Clicking the accession of a particular functional characterization experiment in the table of the “Experiments section” will take you to the summary page of that experiment. It provides further information on the associated metadata and links to the various experimental components.

- The “Control Experiments” section (**Supplementary Fig. 10E**) lists the accessions of all auxiliary and control experiments that belong to the experiment series. The first column in this section provides the accessions of the control experiments, followed by the control type column. The control experiments have different columns in comparison with the columns of the table in the Experiments section. The descriptive biosample summary is applicable in one (ENCSR692ZUM), but not in the other (ENCSR206MVL) control experiment. ENCSR206MVL is an auxiliary experiment that contains the sequencing result of the cloned sgRNA library. There is no biosample associated with this auxiliary experiment, hence the empty biosample summary. ENCSR692ZUM represents the “base-line” CRISPR screen control experiment and its biosample summary is provided. The lab and status columns provide information for each of the control experiments in the series.

- The “Files” section (**Supplementary Fig. 10F**) is the final section of the experiment series summary page. This section is divided into three tabs: Genome browser, Association graph, and File details.

- The “Genome browser” tab (**Supplementary Fig. 11A**) provides rapid visualization of tracks using the embedded Valis genome browser. All visualizable tracks are shown by default. Visualized on the top is the reference genome assembly (GRCh38) followed by the gene tracks (GENCODE V29) and a track with the SNPs reported in The Single Nucleotide Polymorphism Database (dbSNP). The next two tracks are ENCODE specific tracks showing the latest version of the representative DNase hypersensitivity sites (rDHSs) and candidate cis regulatory elements (cCREs) that are a result of integrative analysis of the ENCODE

consortium data. The next track visualizes the guide RNAs locations within the examined region with functional elements specified in the Elements reference dataset (ENCSR827WZZ) associated with the functional experiments in the series. The last two tracks in the genome browser visualize the perturbation signal from the two Flow-FISH CRISPR screens included in this series.

- The "Association graph" tab (**Supplementary Fig. 11B**) displays the data provenance and derivation of the various processed files. The nodes in the graph are clickable and display more information about the node. The yellow nodes represent files, while the blue nodes represent steps in the computational analysis. Click on the yellow node to view the file's accession and other metadata such as file type, output type, mapping assembly, lab and submission date. Click on the blue node to view information about the relevant pipeline analysis step, the step type, the inputs and outputs, and the software used.

- The "Files details" tab contains several collapsible sections (**Supplementary Fig. 11C**). Each section lists data files that are associated with the dataset. The files in each section are presented in a table with information about the file's accession and other metadata such as file type, output type, mapping assembly, size and submission date. Each file is presented in a separate row. A small download icon next to each file accession allows users to download a single file at a time. The collapsible section with the ENCAN792OTU identifier contains files originating from the collective analyses of the experiments in the series. The sections with the experiment identifiers (ENCSR476KHP, ENCSR211TEA, ENCSR206MVL and ENCSR692ZUM) lists files from the corresponding datasets included in this series. The "Reference data" section contains files that capture the investigated loci information.

Supplementary Section 3. Validation of CREs identified from CRISPR screens

High-throughput screening approaches enable perturbations of thousands of cCREs but can suffer from false positives due to technical limitations including cell number, low sequencing depth, and variability between replicates. As such, it is critical to validate hits with individual perturbations and assess whether the validation supports the screen result.

To this end, CREs identified in ENCODE CRISPR screens have been validated via individual sgRNA perturbations to regulate the phenotype used in the respective screen using RT-qPCR and growth competition assays[9,10,12,17,37]. Similarly, CRE-phenotype connections from external datasets have been confirmed using RT-qPCR in addition to more complex characterization methods, including siRNA perturbation of promoter CREs to rule out secondary effects of perturbations leading to changes in gene expression[16], drug resistance assays[29], and excising the CRE in vivo[11].

At minimum, individual sgRNAs should be delivered to the same cell line used in the screen to confirm the perturbation's effect on the screening phenotype is reproducible. Since screens are often performed in clonally-derived cell lines, effects of individual perturbations may be clone-specific. To confirm the change in phenotype is not clone-specific, we recommend delivering the sgRNA(s) to at least one other clonally-derived cell line expressing the same effector, a polyclonal cell line expressing the effector, or in combination with the effector to an unmodified cell line, and measuring the screening phenotype. For FACS-based readouts, when possible it is also advised to confirm the effect on the phenotype using an alternative characterization method, such as antibody staining for a screen performed with an endogenously tagged gene. Finally, orthogonal editing methods can be used to further confirm the CRE-phenotype connection. For example, a CRE-gene connection identified using CRISPRi could be confirmed by excising the CRE via Cas9-paired sgRNA deletion. Alternatively, if the CRE sequence is sufficiently conserved in other model organisms (e.g. mice), the region can be perturbed in vivo with more complex downstream phenotypic characterization.

Supplementary Section 4. Design of sgRNA libraries targeting all ENCODE SCREEN cCREs

To generate sgRNA libraries targeting all human and mouse ENCODE SCREEN v4 cCREs, agnostic of cell type, we first constructed genome-wide GuideScan2 databases for the most recent hg38 and mm10 patches, excluding alternative chromosomes in our analysis. This resulted in two BAM databases containing off-target information, cutting efficiency and specificity scores. The hg38 BAM database is 132 GB in size with 142 million sgRNAs. The mm10 BAM database is 122 GB in size with 135 million sgRNAs. Both databases are available for download online at: https://guidescan.com/downloads.

Next, for each organism and cCRE type, we downloaded the respective cCRE region BED files from the ENCODE SCREEN v4 Registry and used a custom data structure to map the GuideScan2-designed sgRNAs to the cCREs. Specifically, we loaded cCREs into a chromosome-indexed interval tree to enable efficient sgRNA to cCRE mapping and iterated through sgRNAs in the BAM files to find their corresponding cCREs, if any, then emitted the relevant information. This procedure was fast, taking $O(n*\log m)$ time, where n is the number of sgRNAs in the BAM file and m is the number of cCREs; in contrast, other approaches, such as with samtools, took $O(nm)$ time. With parallelization, we could build the database in <2 hours.

Without any filtering based on sgRNA features, there was a median of 26 sgRNAs per cCRE for human proximal enhancer-like signature (pELS) cCREs and fewer for the other cCRE types (**Supplementary Fig. 2 and Supplementary Table 15**). In addition to the unfiltered, genome-wide cCRE sgRNA libraries, we constructed a filtered version of each based on our guidelines that is amenable to either phenotypic (e.g. proliferation) or transcriptional screening readouts (e.g. HCR-FlowFISH). This filtered version removes sgRNAs with a 'TTTT' sequence or a GuideScan2-aggregated CFD specificity score <0.2. For each sgRNA, we computed the distance to its nearest cCRE center, as a proxy for the DHS summit. The sgRNA position was considered the position three nucleotides away from the PAM. As the ENCODE SCREEN cCRE regions are defined by the underlying accessibility signal, and given that DHS signal varies across biosamples even at shared peaks, the center of each cCRE was considered a reasonable cell type-agnostic proxy for the DHS summit. We then sorted sgRNAs by their distance from the cCRE center and selected the closest 20 sgRNAs for each cCRE. This list of filtered and sorted sgRNAs can be downloaded as CSVs from: Guidescan2 ENCODE Results. The source code for this pipeline can be found at: https://github.com/schmidt73/encode_pipeline.

Supplementary Section 5. Standardized non-coding CRISPR Screen File Formats

As we found important differences in screen datasets, the ENCODE CRISPR working group has established uniform file formats to standardize sgRNA design, reporting, and analysis, for CRISPR screens. Six data standards are proposed for future experiments, each incorporating crucial CRISPR screen-specific information into existing standard file formats. The minimally required columns capture the necessary and sufficient parameters for interpreting and analyzing a screen, and can flexibly compare sgRNAs from one screen to the next even if there are differences in the guide spacer design. Three files are proposed to describe data at the sgRNA level: A) ***guide locations*** - a .bed/bigBed visualizable file that describes PAM coordinates, B) ***guide quantifications*** - a .tsv minimally processed file with sequencing counts for each guide in a single sequenced sample, and C) ***perturbation signal*** - a .bigWig visualizable file of fully processed guide data displaying effect sizes of individual sgRNAs. Another set of files describe the data at the CRE level: A) ***element quantifications*** - a .bed+ file describing CREs measured in the screen with additional information such as effect sizes and contributing sgRNAs, B, C) ***element gene interactions signal*** and ***element gene interactions P value*** - .bigInteract visualizable files linking cCRE to target gene (if known) with effect size or significance (P value) of interaction. Full specifications of the file formats are provided in **Supplementary Tables 17-18**. Adopting these standardized file formats as a field will enable cross-screen comparison, streamline meta-analyses, and improve overall usability of the data, and as more non-coding screen data becomes available, our standards will accelerate the iterative pace of analysis and discovery.

Supplementary Section 6. Processed non-coding CRISPR screen file formats

The six standardized data file formats used in this study are detailed below and provide references and quantification at the individual sgRNA-level and CRE-level.

- Guide_location
  - `.bed` of guide coordinates, and corresponding `.bb`, should be bed6
  - Name and score are optional.
  - Includes all regions targeted in an experiment, i.e. include non-significant regions.
  - One per element reference file.
- Guide_quantification
  - `.tsv`, file format specified in **Supplementary Table 16**.
  - This is a minimally processed CRISPR specific datatype meant to collapse fastqs down to the guide-level, enabling cross experiment meta analyses.
  - One per fastq file.
- Perturbation_signal
  - `.bw`, base pair perturbation signal.
  - This is a fully processed file meant for visualization. Use the z-transformed $\log_2$FC for signal values, with 1st bp ('N' in NGG) as location to plot value. On rare occasions with two values at one base pair, average the values.
  - One per replicate per series.
- Element_quantification
  - `.bed`, bed3 with custom additional columns specified in **Supplementary Table 18**.
  - Validated from `.as` file.
  - This is a fully processed file type meant to represent significant CREs.
  - One per series (for replicating peaks) or one per replicate.
- Element_gene_interaction_signal
  - `.bigInteract`, links CRE (from `Element_quantification` file to target gene (if known) with effect size)
  - This is a fully processed file type meant to visualize the `Element_quantification` file.
  - One per series.
- Element_gene_interaction_pvalue
  - `.bigInteract`, links CRE (from `Element_quantification` file to target gene, if known, with P value)
  - This is a fully processed file type meant to visualize the `Element_quantification` file.
  - One per series.

Supplementary Section 7. Expression-based non-coding CRISPR screening

The HCR-flowFISH[9] and FlowFISH[12] protocols can be used for multiple experimental strategies, including single-guide CRISPRi screening, single and paired deletion screening, cellular phenotyping, and different Ccas9 modalities. For HCR-FlowFISH screens, we generally target the gene of interest with highest sensitivity Alexa 647 fluorophores. We also target a control transcript (TBP) with Alexa 488 in order to control for factors such as cell size, RNA abundance, and permeabilization. These experiments are performed using an inducible CRISPRi-BFP line.

More probe pairs usually increases signal to noise[9]. For >100 TPM genes 5+ probe pairs have proven sufficient, while for lower expression targets (~20 TPM) we aim for 40+ probe pairs. For very low expressed targets or short transcripts with few designable probes, higher probe concentration, more amplification time, or both can be effective for detection[9]. Control experiments to detect signal above a negative control (such as EGFP transcripts in cells without the gene), should be performed for any new target.

Multiple sorting strategies were used across the experiments described here, but they generally followed a similar same approach. Prior to sorting, cells are diluted to 1-3x10^8 cell/ml in PBS with 0.5% BSA and filtered using a 30-μm filter (CellTrics, Catalog number 04–004-2326). Cells are first gated to identify single, alive cells that express the CRISPR components. Cells can be sorted into multiple bins based on the fluorescence intensity of the target gene normalized to the control gene. Ratiometric sorting of a target's fluorescence compared to a reference marker's fluorescence increases precision and better-discriminates signal from cell-intrinsic noise[62]. On the Astrios control software (Summit v6.3.1) one can use the color compensation tool to subtract a portion of each cell's AF647 signal based on the intensity of its AF488 signal. On the Sony MA-900 sorter, one can compensate the APC-A vs FITC-A signals to generate compensated APC histograms. We then gate on the highest 10% and lowest 10% of the resulting population for 2-way sorting, or use 0–10, 10–20, 35–45, 55–65, 80–90, and 90–100% bins for 6-way sorting. As a standard guideline, we aim for each bin to have a total of cells equal to 100X guide coverage, however more coverage can reduce noise.

Binning decisions are typically constrained by the sorter's capabilities, but these decisions affect downstream analysis steps and the information obtained in the experiment. The effect of bin number and width has been modeled and discussed[62,63]. As a conceptual summary, the narrower a bin is, the more information there is about the true effect value of a sgRNA that ends up in it. With infinite bins, a guide's exact value is known; on the other extreme, a single bin boundary would only suggest whether a sample's value is above or below that threshold–sufficient to estimate an effect, but with poor estimation accuracy. Simulating a practical experiment, de Boer et al. find an improvement going from two to four, or four to six bins, but with rapidly diminishing returns. Peterman and Levine also observe similar improvements in estimation accuracy beyond two bins. The number and width of bins must also consider the total coverage of the distribution; if only a small part of the distribution is captured by the bins, many of the potential guides–and information–is lost. Although the information from each measured guide is higher, the net result is a loss of information. This is recapitulated in simulations showing losses in estimation accuracy with bins capturing less than 20~25% of the distribution[63,64]. Bins that are similar or larger in size to the distribution lead to large estimation biases as they carry little, if any, information–especially if a single bin encompasses the entire distribution of a variant's measured effects[62]. For a population whose measured effects follow a continuous distribution (i.e. are not binary), bins often begin from the edges of the distribution; where more than two bins are used, bin boundaries are typically continuous with the neighboring bin(s) from their respective edges (as in de Boer et al.'s simulations), or symmetrical across the distribution. Evaluating the dynamic ranges and distributions

of positive and negative controls–and the screening library–by flow cytometry can inform the optimal distribution of gates according to the conceptual guidelines discussed here and in the referenced simulation studies.

Supplementary Figures

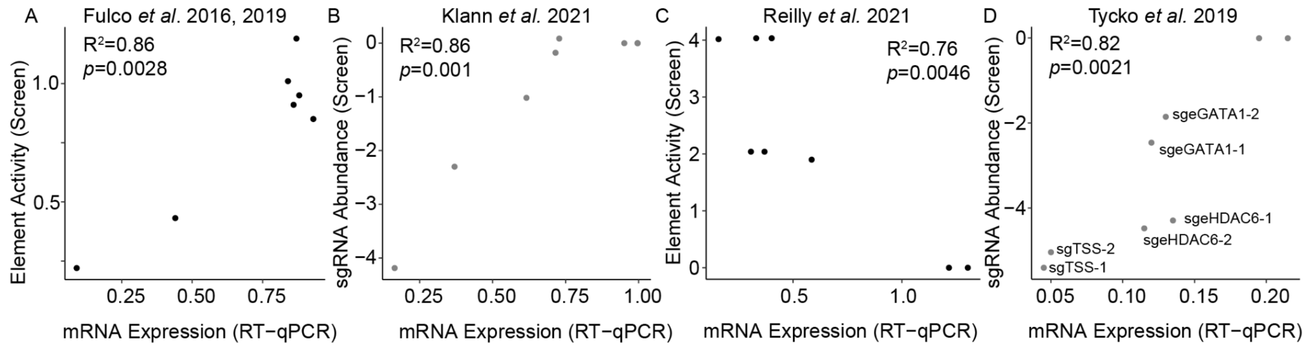

Supplementary Fig. 1. Individual sgRNA validations correlate with non-coding CRISPR screen results

A) Element-level score from FlowFISH screen in *GATA1* locus versus *GATA1* mRNA expression. A lower element activity value (y-axis) indicates the regulatory element exhibited greater function in the experiment. **B**) Validations targeting distal enhancer of LMO2 identified in growth screen targeting all DHSs in K562s[37]. Abundance of sgRNAs versus LMO2 mRNA expression. A lower sgRNA abundance indicates the effect of the perturbation was more detrimental to cell growth, and the perturbed element exhibited greater regulatory function. **C**) Element-level score from FlowFISH screen in FADS1/3 locus versus FADS3 mRNA expression[9]. A greater element activity score indicates the regulatory element exhibited greater function in the experiment. **D**) Abundance of sgRNAs tiling the *GATA1* locus versus *GATA1* mRNA expression[17]. sgRNA labels next to each point correspond to labels in **Fig. 5** and **Supplementary Table 14**. A lower sgRNA abundance indicates the effect of the perturbation was more detrimental to cell growth, and the perturbed element exhibited greater regulatory function. **A-D**) All validations were performed using lentiviral transduction of individual sgRNAs into dCas9^{KRAB}-expressing K562 cells (Two-sided Pearson correlation values and P values calculated across biological replicates noted within each panel; black and gray indicate FACS-based and growth-based screens, respectively). For **A**,**C**,**D**, each point is the mean of two biological replicates. For **B**, each point is the mean of three biological replicates.

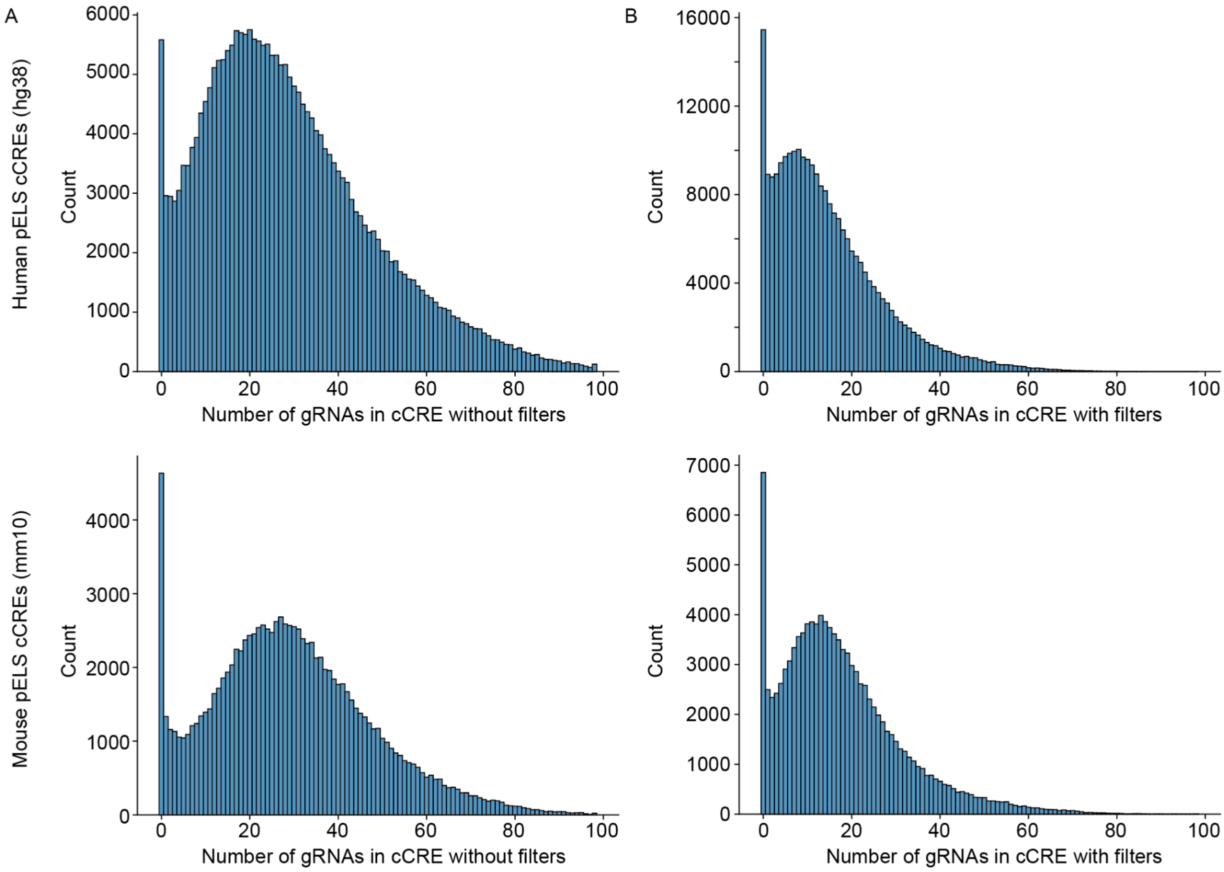

Supplementary Fig. 2. GuideScan2-designed sgRNAs targeting all cCREs from the ENCODE SCREEN portal.

A) The distribution of GuideScan2-designed sgRNAs per cCRE in the ENCODE SCREEN Registry v4 proximal enhancer-like signature (pELS) cCRE sets for human (top) and mouse (bottom) without and **B**) with filters to remove sgRNAs with a ‘TTTT’ sequence or GuideScan2-aggregated CFD specificity score<0.2.

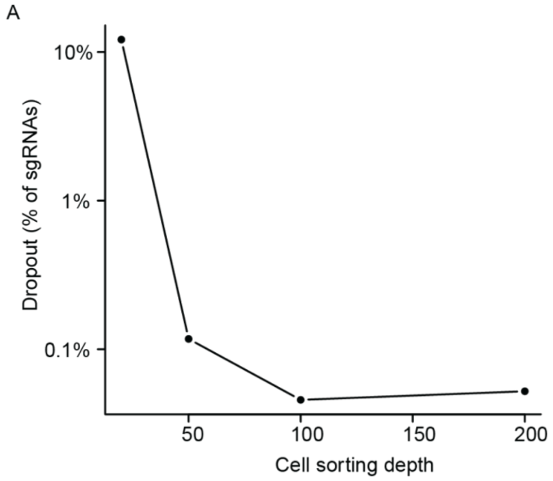

Supplementary Fig. 3: Guide drop out rate with varying HCR-FlowFISH sorting depths

A) sgRNA dropout rates (sgRNA with <10 mapped reads for low- or high-expression sorting bins) for varying cell sorting depth (20x, 50x, 100x, 200x) for CRISPRi *GATA1* HCR-FlowFISH performed at 2000x sequencing depth (n=1 replicate)

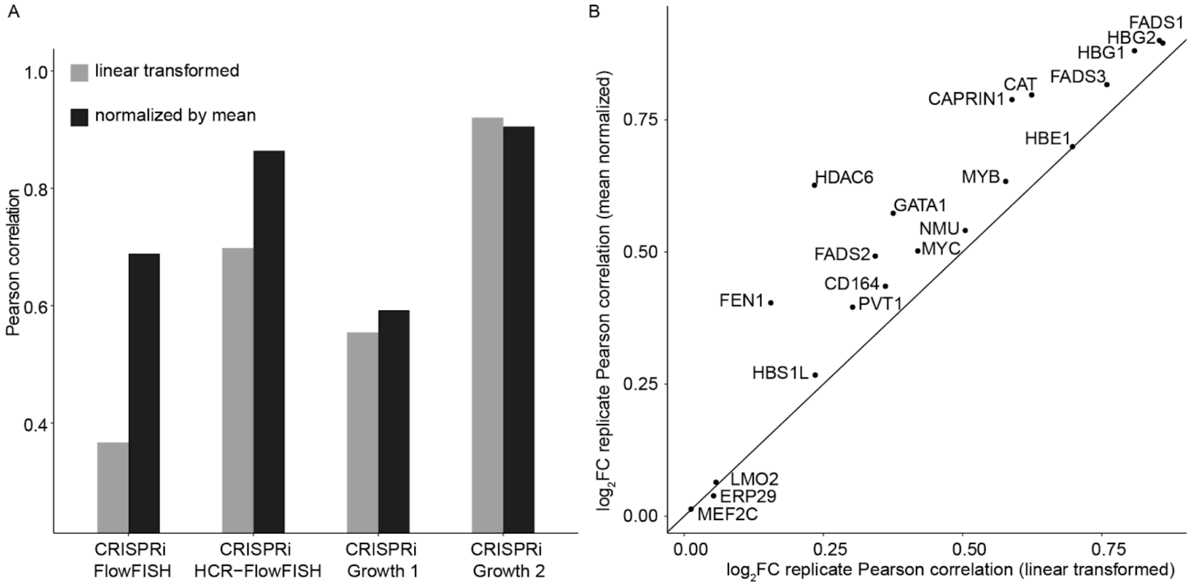

Supplementary Fig. 4: Effect size normalization methods affect biological replicate correlation

A) Comparison of GATA1 tiling screens with linear transformed or mean-normalized effect size calculations (**Methods**). The guide-wise Pearson correlation of log_2FC between two bio-replicates is shown for the different normalization methods (n=167 sgRNAs commonly used across the GATA1 screens with GuideScan specificity >0.2). **B**) The guide-wise Pearson correlations of log_2FC between two bio-replicates for each of the 20 genes screened using HCR-FlowFISH, using mean normalized (y-axis) and linear transformed (x-axis) log_2FC.

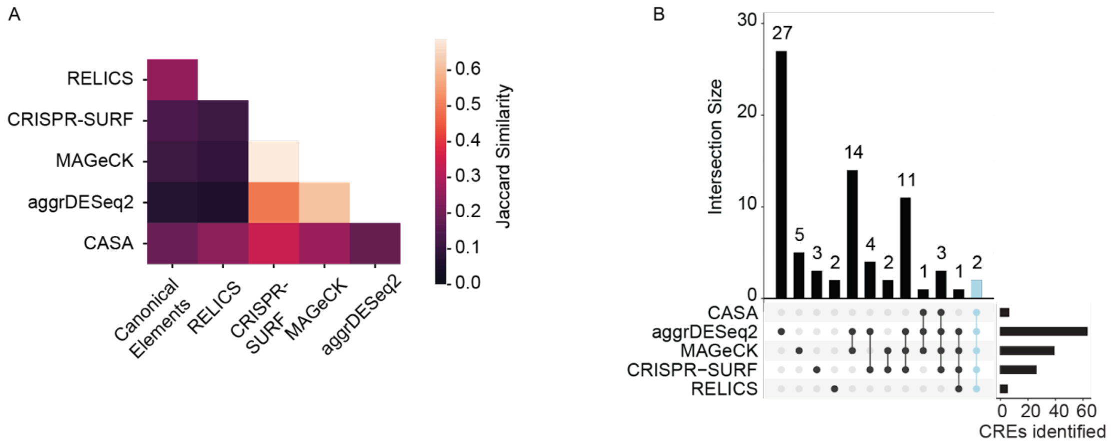

Supplementary Fig. 5. Overlap between peak calls on specificity-filtered CRISPRi tiling screen of *GATA1* locus

A) Quantification of pairwise overlap of peak calls by Jaccard Similarity. High values (lighter colors) correspond to greater overlap. **B**) Upset plot of intersections of peaks identified by the 5 peak callers.

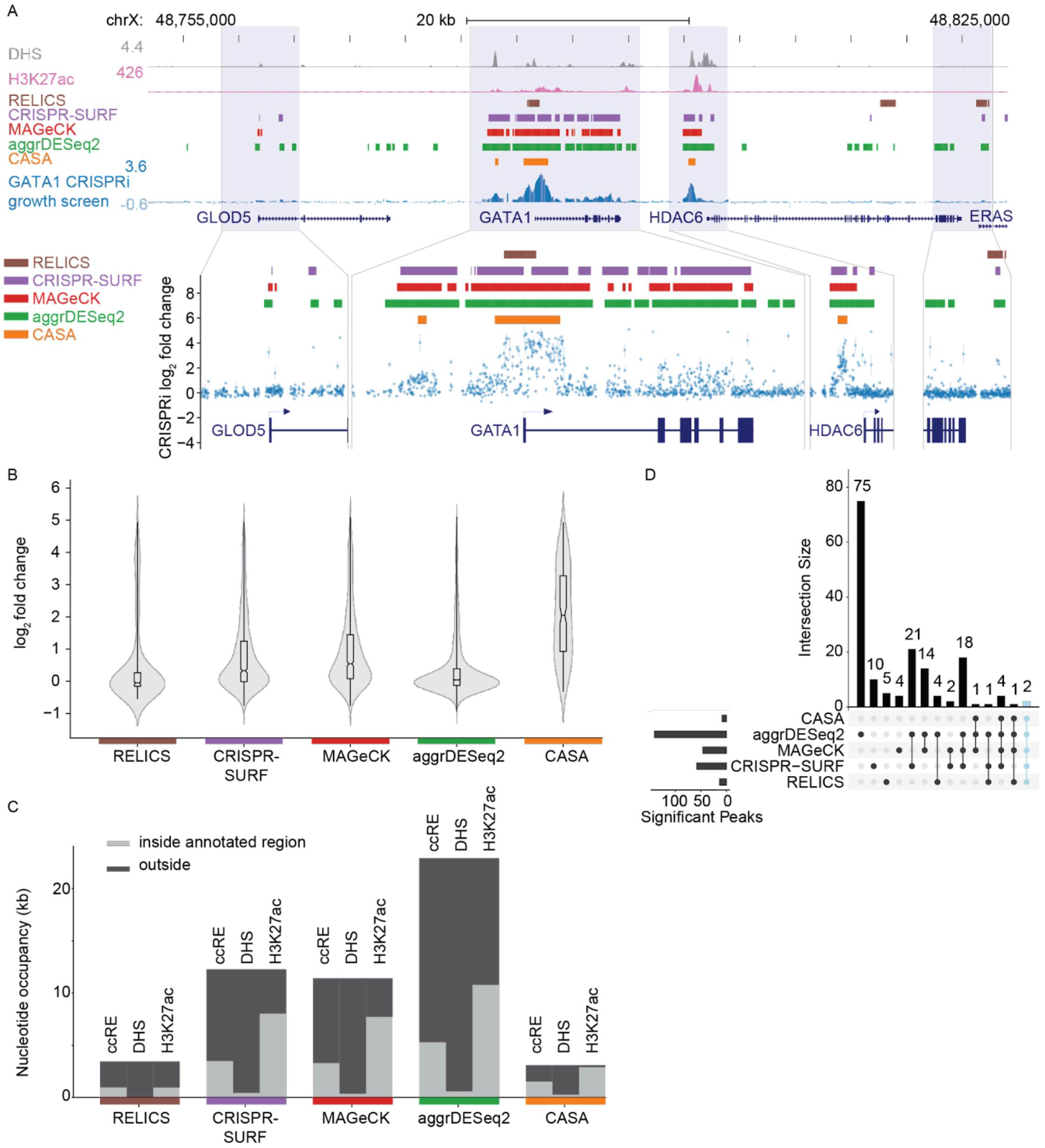

Supplementary Fig. 6. Peak calls without filtering out low specificity sgRNAs

A) sgRNA mediated growth effects (blue), H3K27ac-ChIP signal (pink), and DNase Hypersensitivity signal (gray) for a CRISPRi growth screen at the GATA1 locus, without removing low specificity sgRNAs. Dense tracks show peak calls using 5 different CRISPR screen analysis tools: CASA (orange), aggrDESeq2 (green), MAGeCK (red), CRISPR-SURF (purple), and RELICS (brown). Zoomed-in regions show individual sgRNA effects (points, mean; bars, min-max range of observations between n=2 biological screening replicates). **B**) Distribution of average sgRNA effects from two experimental replicates for sgRNAs falling within peaks identified by different CRISPR screen analysis tools (center line, median; notch confidence interval of the median; box limits, first and third quartiles; whiskers range of all data points; violin, kernel density estimation; n= 302, 955, 829, 2579, 222 sgRNAs in CREs from left to right; Welch's two-tailed t-test vs shuffled $-\log_{10}p$= 7.7, 57.4, 71.5, 38.8, 56.7 from left to right). **C**) Total peak area inside (light gray) and outside (dark gray) of annotated chromatin features for each peak caller. **D**) Intersections of peaks identified by the 5 peak callers.

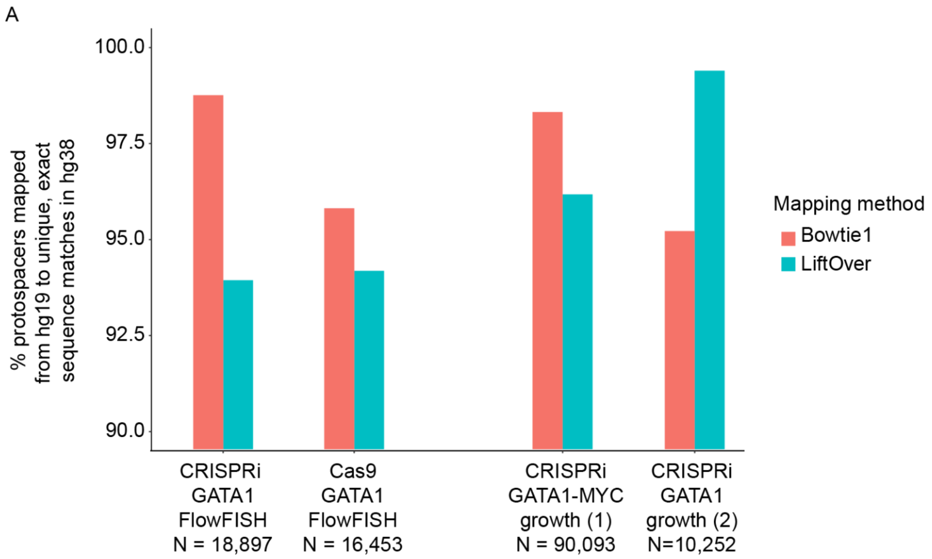

Supplementary Fig. 7. Mapping sgRNAs to reference genome for data standardization

A) Four CRISPR screen sgRNA libraries in hg19 were mapped to hg38 using bowtie1 and LiftOver. For three out of the four libraries, bowtie1 resulted in higher rates of unique and exact sequence mapping (**Supplementary Section 1**). CRISPRi-Growth datasets are (1) Tycko *et. al.* 2019 and (2) Fulco *et. al.* 2019.

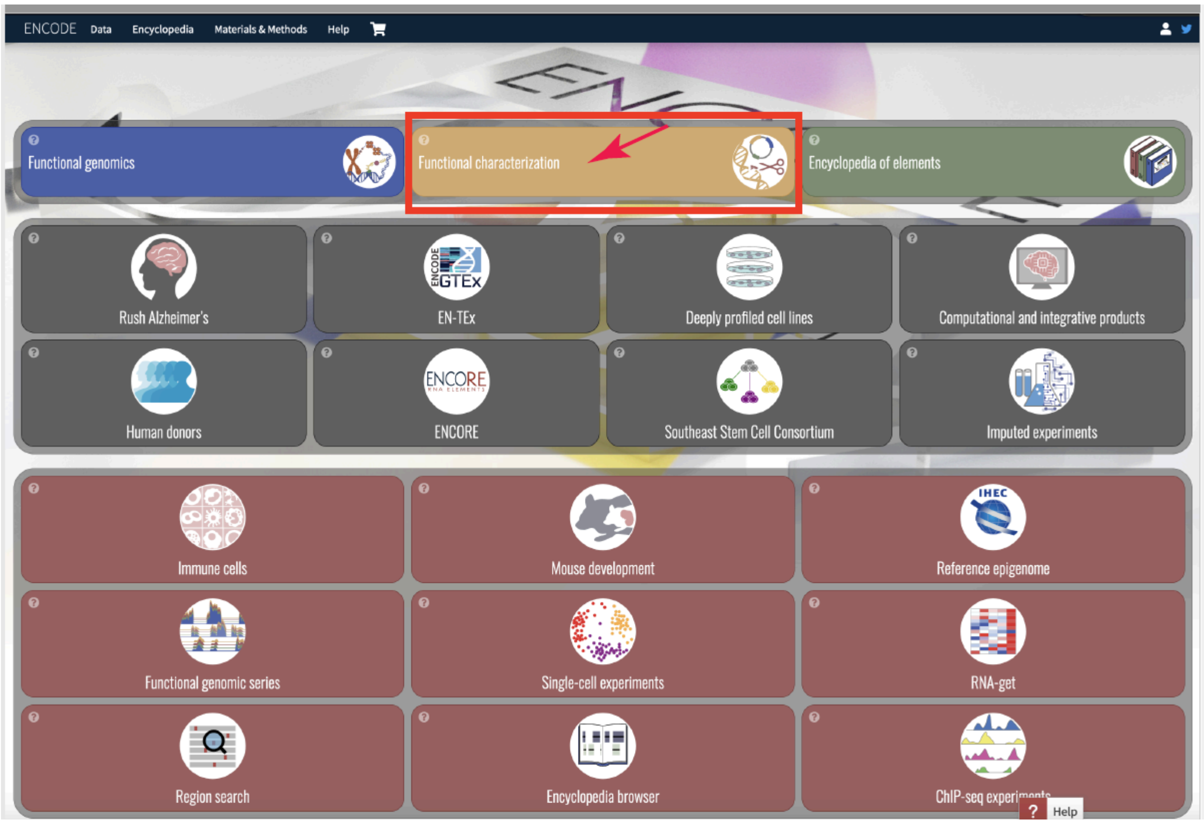

Supplementary Fig. 8. 'Functional Characterization' card on the ENCODE home page

Representative image of the ENCODE portal home page. The red box indicates the card to select to navigate to the Functional Characterization assay datasets.

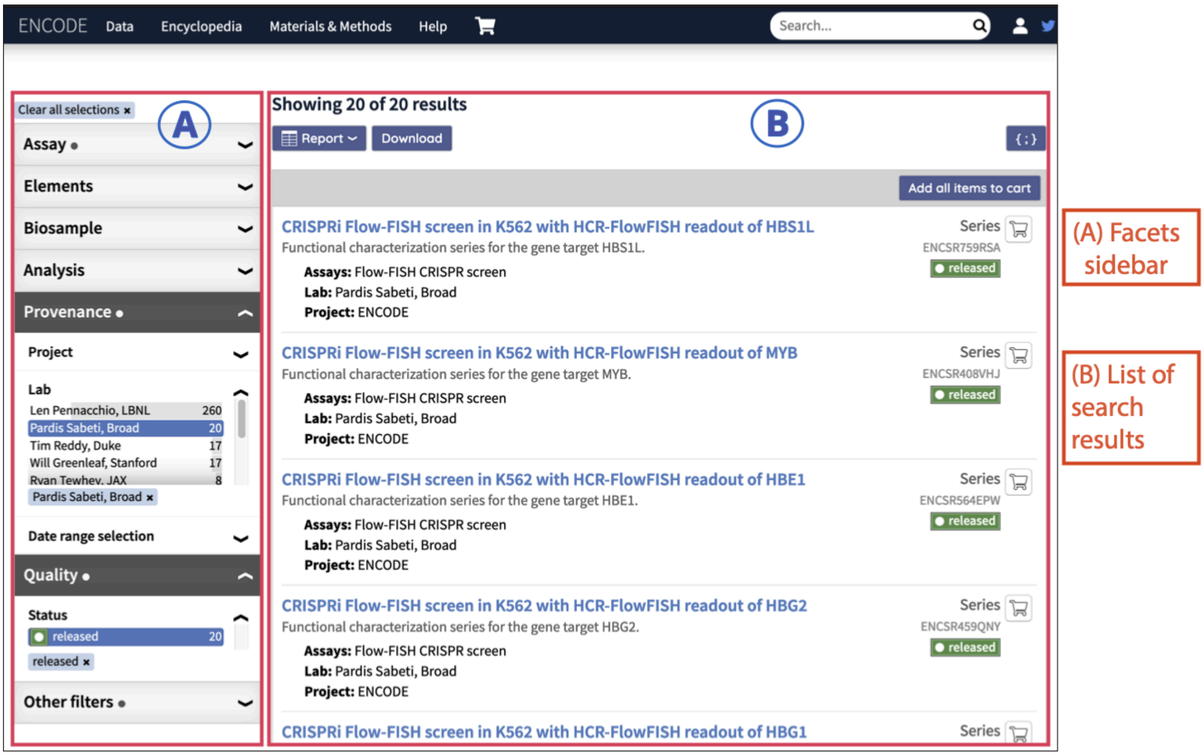

Supplementary Fig. 9. Filtering search results in the ENCODE portal

A) A view of the facets sidebar with the items that should be selected and the list of the resulting ENCODE Functional Characterization Series. **B**) Each Series is shown with a brief summary of the biological material, assay name, and a link to its individual experiment series summary page with more metadata details. The second Series from the list (ENCSR408VHJ) is selected as an example and further exploration.

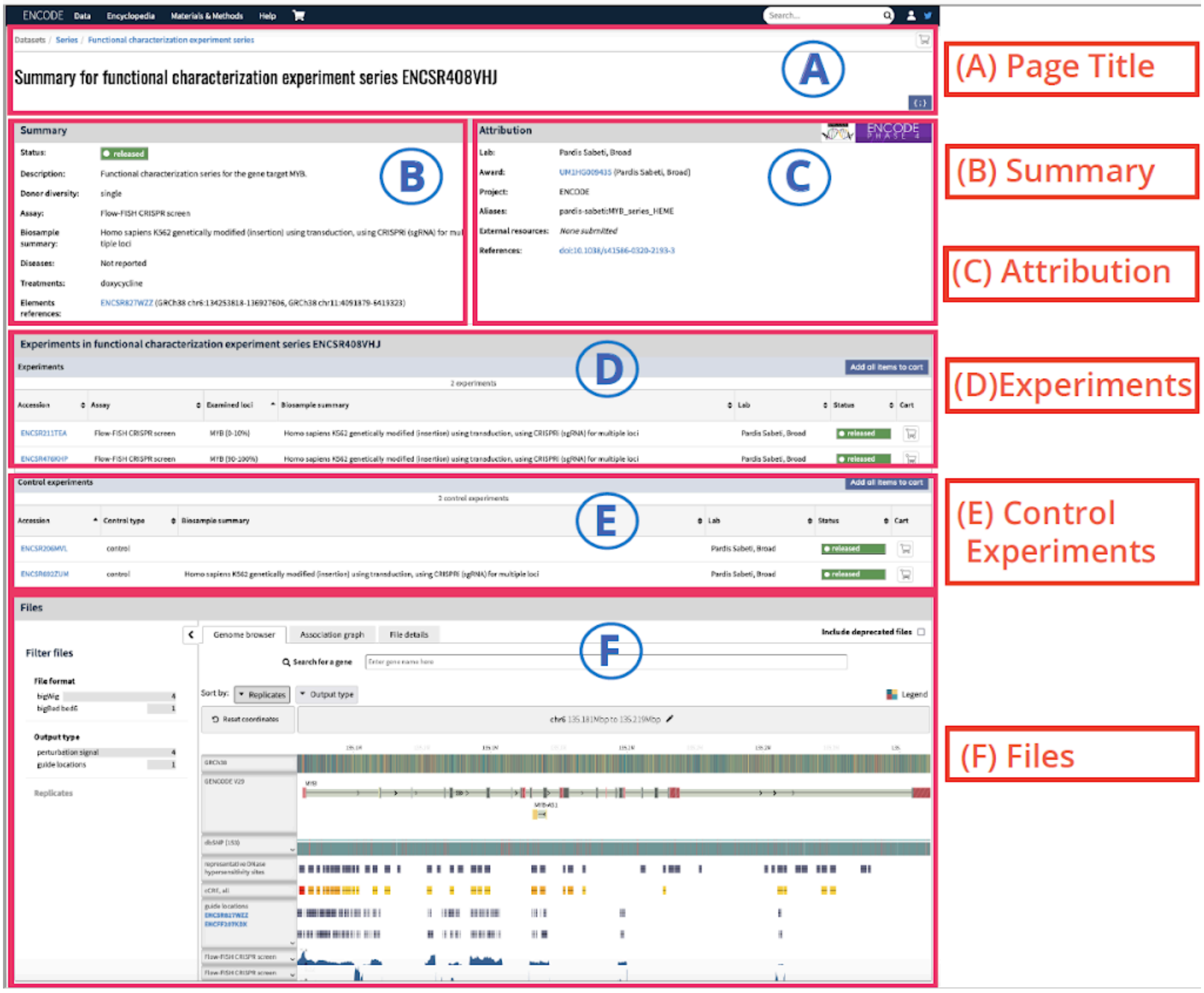

Supplementary Fig. 10. An experiment series summary page

The page is organized into six distinct sections. **A**) Page Title: providing the series title and accession. **B**) Summary: providing key information about the series. **C**) Attribution: providing information about the lab that performed the experiment, aliases and external resources. **D**) Experiments: providing information about all experiments that have been included in the series. **E**) Control Experiments: providing information about all auxiliary and control experiments that have been included in the series. **F**) Files: contains information about the files associated with the series.

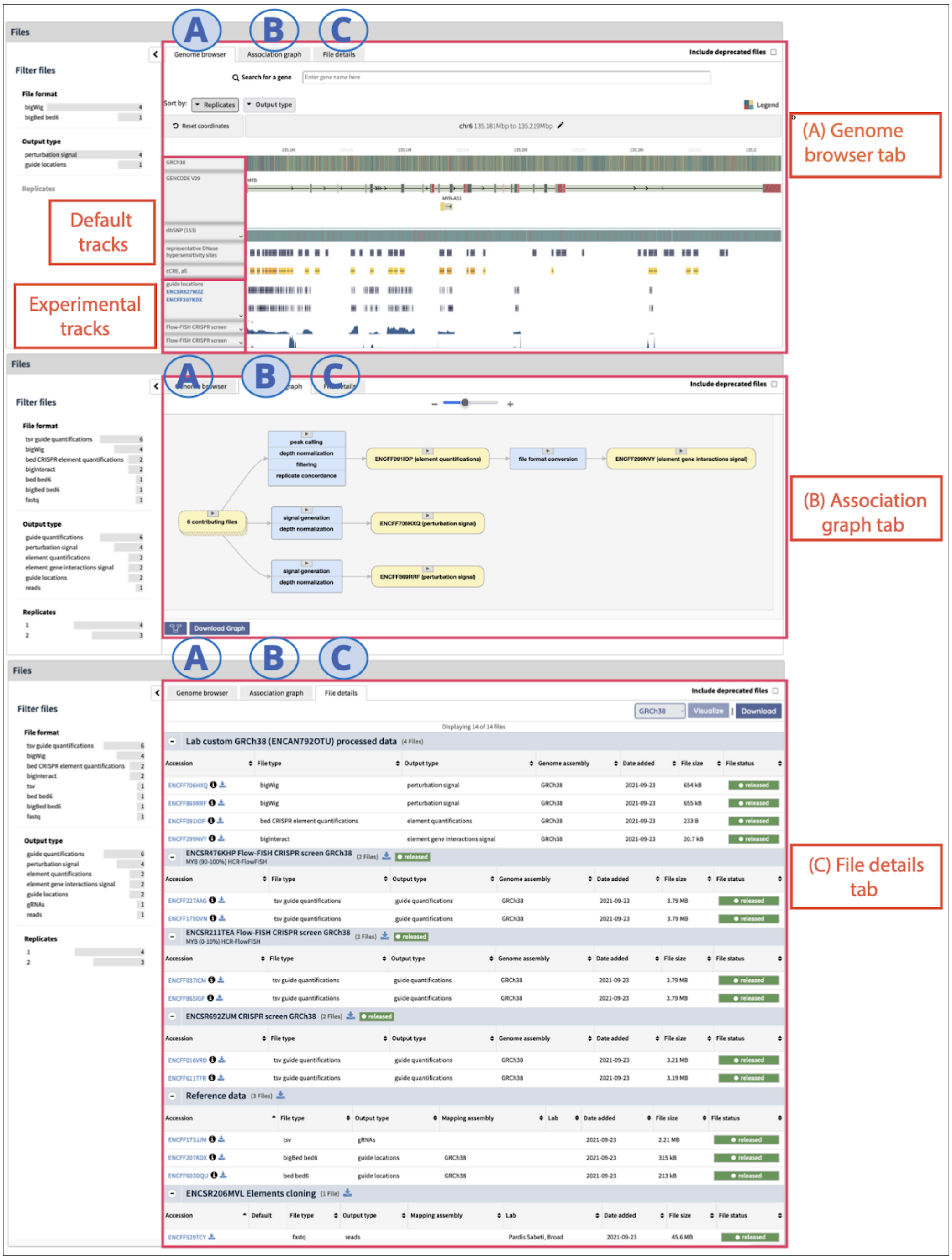

Supplementary Fig. 11. The 'Files Section' of the experiment series summary page

The Files Section of the experiment series summary page contains three tabs. **A**) Genome browser tab: visualizes tracks using the embedded Valis genome browser. **B**) Association graph tab: displays the data provenance and derivation of downstream processed files. **C**) File details tab: lists the files that are associated with the series.

Supplementary Tables

A description of the contents of all Supplementary Tables is provided below. **Supplementary Tables 1-12** and **13-18** are provided in one separate file. **Supplementary Table 12** is a separate Excel file.

Supplementary Table 1: Bulk CRISPR screens in the ENCODE data portal.
Supplementary Table 2: CREs identified from available CRISPR screens performed in human biosamples.
Supplementary Table 3: Overview of noncoding CRISPR screen approaches.
Supplementary Table 4: Annotation files used in K562 meta analysis.
Supplementary Table 5: CREs identified from available CRISPR screens performed in K562s.
Supplementary Table 6: Fisher's exact test results for enrichment of cell-type agnostic and K562 annotations in functional regulatory elements.
Supplementary Table 7: Signal values and test results for comparison of K562 feature signal in significant CREs versus perturbed regions.
Supplementary Table 8: Overlap of CREs with accessible chromatin and H3K27ac in multiple cell types.
Supplementary Table 9: Negative control sgRNAs used in this study.
Supplementary Table 10: hg38 Safe-targeting sgRNA library.
Supplementary Table 11: Summary of common sgRNA design tools.
Supplementary Table 12: sgRNA library and read counts for *GATA1* titration experiments.
Supplementary Table 13: Summary of analysis tools used in peak calling comparison.
Supplementary Table 14: *GATA1* locus sgRNAs for validations.
Supplementary Table 15: GuideScan2 sgRNA counts at SCREEN cCREs.
Supplementary Table 16: CRISPR screen guide_quantification file format and contents.
Supplementary Table 17: CRISPR screen element_quantification file format and contents.
Supplementary Table 18: Accession IDs from ENCODE portal used in this study.

Public datasets accessed

Accession IDs for public datasets used in this study are provided in **Supplementary Table 18**.

Author contributions

S.K.R., J.T., D.Y., and A.K. conceived of the study. S.K.R., D.Y., J.T., J.W.O., L.R.B., S.J.G., L.L., A.M-S., B.R.D., and X.R. analyzed data. A.M-S. performed GATA1 HCR FlowFISH coverage titration experiments. T.G. and K.S. performed the Gitr T-reg screen. I.G., D.Y., L.R.B., J.W.O., and Y.L. curated and designed the ENCODE CRISPR screening portal, and S.K.R., D.Y., A.M-S., J.W.O., L.R.B., J.M.E., I.G., and Y.L. developed the file formats. J.W.O. and A.M-S. generated public repositories to visualize CRISPR screen data and results. J.W.O. and L.R.B. generated public repository for all code used for analyses in the paper. I.G. wrote the tutorial for navigating screening data on the ENCODE portal. L.R.B. performed literature review for design tools and analysis methods. H.S., D.Y., J.T., J.M., C.L., and Y.P. designed the genome-wide ENCODE SCREEN cCRE sgRNA libraries. M.A.B. advised analyses. S.K.R., D.Y., J.T., J.W.O., L.R.B., S.J.G., L.L., A.M-S., B.R.D., X.R., K.G., A.D.W., and J.M.E. wrote the paper, with revisions from all authors. S.K.R., M.C.B., M.A.B., J.M.E., A.K., C.A.G, and T.E.R supervised and developed the project. M.C.B., M.A.B., W.J.G., C.A.G., A.K., T.E.R., P.C.S., and Y.S. acquired funding.

The authors would like to note that when reporting this publication, all co-first authors have agreed that co-listed authors can be listed in any order, including arranging themselves first to best highlight the equal contribution. AMS, BRD, IG, and HS, contributed equally to this work

Author contributions

S.K.R., J.T., D.Y., and A.K. conceived of the study. S.K.R., D.Y., J.T., J.W.O., L.R.B., S.J.G., L.L., A.M-S., B.R.D., A.B., and X.R. analyzed data. A.M-S. performed GATA1 HCR FlowFISH coverage titration experiments. T.G. and K.S. performed the Gitr T-reg screen. I.G., D.Y., I.Y, K.A, S.K.R., L.R.B., J.W.O., and Y.L. curated and designed the ENCODE CRISPR screening portal, and S.K.R., D.Y., A.M-S., J.W.O., L.R.B., J.M.E., I.G., and Y.L. developed the file formats. J.W.O. and A.M-S. generated public repositories to visualize CRISPR screen data and results. J.W.O. and L.R.B. generated public repository for all code used for analyses in the paper. I.G. wrote the tutorial for navigating screening data on the ENCODE portal. L.R.B. performed literature review for design tools and analysis methods. H.S., D.Y., J.T., J.M., C.L., and Y.P. designed the genome-wide ENCODE SCREEN cCRE sgRNA libraries. M.A.B. advised analyses. S.K.R., D.Y., J.T., J.W.O., L.R.B., S.J.G., L.L., A.M-S., B.R.D., X.R., K.G., A.D.W., and J.M.E. wrote the paper, with revisions from all authors. S.K.R., M.C.B., M.A.B., J.M.E., A.K., C.A.G, and T.E.R supervised and developed the project. M.C.B., M.A.B., W.J.G., C.A.G., A.K., T.E.R., P.C.S., and Y.S. acquired funding.
The authors would like to note that when reporting this publication, all co-first authors have agreed that co-listed authors can be listed in any order, including arranging themselves first to best highlight the equal contribution. AMS, BRD, IG, and HS, contributed equally to this work

Competing Interests Statement

A.K. is scientific co-founder of Ravel Biotechnology, is on the scientific advisory board of PatchBio, SerImmune, AINovo, TensorBio and OpenTargets, is a consultant with Illumina and owns shares in DeepGenomics, Immuni and Freenome. C.A.G. is a co-founder of Tune Therapeutics and Locus Biosciences, and an advisor to Tune Therapeutics and Sarepta Therapeutics. C.A.G. is an inventor on patents and patent applications related to CRISPR epigenome editing. J.T. and M.C.B. acknowledge an outside interest in Stylus Medicine. L.L. is currently employed by Sana Biotechnology. D.Y. is currently employed by Amber Bio. P.C.S is a co-founder of and consultant to

Sherlock Biosciences and Board Member of Danaher Corporation. She is a shareholder in both companies. W.J.G. is a co-founder of Epinomics and an adviser to 10X Genomics, Guardant Health and Centrillion. The remaining authors declare no competing interests.

Supplementary References

62. Peterman, N. & Levine, E. Sort-seq under the hood: implications of design choices on large-scale characterization of sequence-function relations. *BMC Genomics* **17**, 206 (2016).
63. de Boer, C. G., Ray, J. P., Hacohen, N. & Regev, A. MAUDE: inferring expression changes in sorting-based CRISPR screens. *Genome Biol.* **21**, 134 (2020).
64. Nagy, T. & Kampmann, M. CRISPulator: a discrete simulation tool for pooled genetic screens. *BMC Bioinformatics* **18**, 347 (2017).
65. Nasser, J. *et al.* Genome-wide enhancer maps link risk variants to disease genes. *Nature* **593**, 238–243 (2021).
66. Corces, M. R. *et al.* An improved ATAC-seq protocol reduces background and enables interrogation of frozen tissues. *Nat. Methods* **14**, 959–962 (2017).
67. Gemberling, M. P. *et al.* Transgenic mice for in vivo epigenome editing with CRISPR-based systems. *Nat. Methods* **18**, 965–974 (2021).
