# Supplementary material for: Multicenter integrated analysis of noncoding CRISPRi screens
Source: Nat Methods. 2024 Mar 19;21(4):723–34. doi: 10.1038/s41592-024-02216-7 (PMC11009116; doi:10.1038/s41592-024-02216-7)
Supplement: Supplementary file 2 — Reporting Summary [file 41592_2024_2216_MOESM2_ESM.pdf]

nature portfolio

Corresponding author(s): Steven K. Reilly, Josh Tycko

Last updated by author(s): Dec 16, 2023

Reporting Summary

Nature Portfolio wishes to improve the reproducibility of the work that we publish. This form provides structure for consistency and transparency in reporting. For further information on Nature Portfolio policies, see our Editorial Policies and the Editorial Policy Checklist.

Statistics

For all statistical analyses, confirm that the following items are present in the figure legend, table legend, main text, or Methods section.

n/a	Confirmed	
☐	☒	The exact sample size (*n*) for each experimental group/condition, given as a discrete number and unit of measurement
☐	☒	A statement on whether measurements were taken from distinct samples or whether the same sample was measured repeatedly
☐	☒	The statistical test(s) used AND whether they are one- or two-sided<br>*Only common tests should be described solely by name; describe more complex techniques in the Methods section.*
☒	☐	A description of all covariates tested
☐	☒	A description of any assumptions or corrections, such as tests of normality and adjustment for multiple comparisons
☐	☒	A full description of the statistical parameters including central tendency (e.g. means) or other basic estimates (e.g. regression coefficient) AND variation (e.g. standard deviation) or associated estimates of uncertainty (e.g. confidence intervals)
☐	☒	For null hypothesis testing, the test statistic (e.g. *F*, *t*, *r*) with confidence intervals, effect sizes, degrees of freedom and *P* value noted<br>*Give P values as exact values whenever suitable.*
☒	☐	For Bayesian analysis, information on the choice of priors and Markov chain Monte Carlo settings
☒	☐	For hierarchical and complex designs, identification of the appropriate level for tests and full reporting of outcomes
☐	☒	Estimates of effect sizes (e.g. Cohen's *d*, Pearson's *r*), indicating how they were calculated

Our web collection on statistics for biologists contains articles on many of the points above.

Software and code

Policy information about availability of computer code

Data collection	No software was used to collect data.
Data analysis	The code for CASA can be found at https://github.com/sjgosai/casa. CASA version 0.2.3 was used; the commit hash corresponding to the code used in the paper is cc7ba944dc866611ef338e68a256005656f4574a. DESeq2 version 1.42 was used. RELICS v2 was used. The code for using GuideScan2 to design sgRNAs for all cCREs can be found at https://github.com/schmidt73/encode_pipeline. The code used for other analyses and to make figures is available online at https://github.com/Reilly-Lab-Yale/ENCODE-CRISPR.

For manuscripts utilizing custom algorithms or software that are central to the research but not yet described in published literature, software must be made available to editors and reviewers. We strongly encourage code deposition in a community repository (e.g. GitHub). See the Nature Portfolio guidelines for submitting code & software for further information.

Data

Policy information about availability of data

All manuscripts must include a data availability statement. This statement should provide the following information, where applicable:

- Accession codes, unique identifiers, or web links for publicly available datasets
- A description of any restrictions on data availability
- For clinical datasets or third party data, please ensure that the statement adheres to our policy

The data is available in the online ENCODE portal. ENCODE accession numbers and other experiment metadata are provided in Supplementary Table 1. The genomic

and epigenomic annotation files used in this analysis are provided in Supplementary Table 4. Accession IDs for public datasets used in this study are provided in Supplementary Table 18.
All CRISPR screen datasets used in this study are available in the online ENCODE portal and accession IDs are included in Supplementary Table 1. sgRNA counts for the GATA1 titration experiments are provided in Supplementary Table 11.

The Gitr T-reg screening data can be found here: https://www.dropbox.com/scl/fo/7q92wt7zyejfkwetsgsr6/h?rlkey=30ytwfaazty33bz3ez30coiy8&dl=0

Public repositories to visualize CRISPR screen data and results from Fig. 1 and Fig. 6 are listed below:
Fig. 1: https://data.cyverse.org/dav-anon/iplant/home/joh27/track_hub_fig1/hub.txt
Fig. 6: https://data.cyverse.org/dav-anon/iplant/home/ohjinwoo94/track_hub_fig6/hub.txt
The hg38 human reference genome was used.

Human research participants

Policy information about studies involving human research participants and Sex and Gender in Research.

Reporting on sex and gender	n/a
Population characteristics	n/a
Recruitment	n/a
Ethics oversight	n/a

Note that full information on the approval of the study protocol must also be provided in the manuscript.

Field-specific reporting

Please select the one below that is the best fit for your research. If you are not sure, read the appropriate sections before making your selection.

☒ Life sciences ☐ Behavioural & social sciences ☐ Ecological, evolutionary & environmental sciences

For a reference copy of the document with all sections, see nature.com/documents/nr-reporting-summary-flat.pdf

Life sciences study design

All studies must disclose on these points even when the disclosure is negative.

Sample size	No sample-size calculation was performed. CRISPR screens were performed with 2 biological replicates that were separately screened and sequenced. Calling hit CREs within these screens relies on the scores of multiple sgRNAs targeting the element (with two bioreps each).
Data exclusions	No data exclusions
Replication	Screens were analyzed with biological replicates and the screen scores for a subset of 30 sgRNAs were confirmed to correspond with individual validation experiments (Supplementary Fig 1). In the GATA1 locus, a similar CRISPRi screen was performed independently by three laboratories and results compared to identify replicable hits.
Randomization	Unbiased screens were performed wherein libraries of targeting and negative control sgRNAs are delivered into cell populations by lentivirus, so control and targeted groups are grown together in the same cell population. No need to select certain samples for certain treatment groups in this context.
Blinding	Blinding was not possible in the context wherein individual researchers were responsible for performing full screen experiments through data generation. However, datasets from each center were analyzed by researchers from another center.

Reporting for specific materials, systems and methods

We require information from authors about some types of materials, experimental systems and methods used in many studies. Here, indicate whether each material, system or method listed is relevant to your study. If you are not sure if a list item applies to your research, read the appropriate section before selecting a response.

March 2021

Materials & experimental systems

n/a	Involved in the study
☒	☐ Antibodies
☐	☒ Eukaryotic cell lines
☒	☐ Palaeontology and archaeology
☒	☐ Animals and other organisms
☒	☐ Clinical data
☒	☐ Dual use research of concern

Methods

n/a	Involved in the study
☒	☐ ChIP-seq
☐	☒ Flow cytometry
☒	☐ MRI-based neuroimaging

Eukaryotic cell lines

Policy information about cell lines and Sex and Gender in Research

Cell line source(s): K562 cells with a doxycycline-inducible CRISPRi were a gift of the Lander lab.

Authentication: Not authenticated. However, CRISPRi-BFP was induced for 24 h with a final concentration of 1 µg/ml doxycycline (VWR) and then active CRISPRi was checked by confirming dox-induced BFP/CRISPRi signal was observed in >90% of cells by flow cytometry.

Mycoplasma contamination: Quarterly mycoplasma testing for the cells used in GATA1 experiments. All cells tested negative for mycoplasma.

Commonly misidentified lines (See ICLAC register): None

Flow Cytometry

Plots

Confirm that:

☒ The axis labels state the marker and fluorochrome used (e.g. CD4-FITC).

☒ The axis scales are clearly visible. Include numbers along axes only for bottom left plot of group (a 'group' is an analysis of identical markers).

☒ All plots are contour plots with outliers or pseudocolor plots.

☒ A numerical value for number of cells or percentage (with statistics) is provided.

Methodology

Sample preparation: Naive CD4+ T cells were harvested from spleen and lymph nodes of Foxp3-eGFP dCas9-KRAB CD4-CRE C576BL/6 mice using magnetic selection (Thermo Cat# 8804-6821-74)67. 4 mice were used as independent biological replicates. Cells were seeded at 0.5e6 cells/mL and cultured in complete RPMI (10% FBS, 1% Penicillin, 1% Streptamycin, 1% Gentamicin, 1% L-glutamine, 1% HEPES, 1% sodium pyruvate, 55nM 2-mercaptoethanol) and activated with Th0 conditions (250 ng/mL αCD3, 1 µg/mL αCD28, 2 µg/mL αIL-4, 2 µg/mL αIFNg). Cells were transduced at 24 hours with viral supernatant containing 6.66ng/µL polybrene and at 900 x g for 2 hours at 30C. Cells were then cultured in Treg polarizing conditions (Th0 conditions + 10ng/mL IL-2, 10ng/mL hTgfβ) for 96 hours. Live cells were stained for viability-e780 (Thermo Cat# 65-0865-14), Gitr-PE (BD Bioscience Cat# 558140), CD4-e450 (Thermo, Cat# 48-0042-80), Thy1.1-APC (Stem Cell Technologies, Cat# 60024AZ) for 30 minutes on ice and sorted using a Sony SH800Z with a 70 µm chip.

Instrument: Sony SH800Z with a 70µm chip

Software: Sony SH800 software was used to collect and analyze the data.

Cell population abundance: We selected the 15% high and low expressing cells for sequencing. The purity of sorting (52-70%) resulted is shown in Extended Data Figure 5C. At least 40,000 cells were sorted from the top and bottom 15% of Gitr signal (Gating: Lymphocytes / Live / Singlets / CD4+ / THY1.1+ / FOXP3-eGFP+ / GITRhi/lo).

Gating strategy: FSC/SSC gates were drawn as a polygon to select viable cells. Linear gates drawn to capture the higher of two clear peaks, and polygon gates drawn to capture the 15% High and Lo cells, used for sorting viable CD4+/Thy1+/Foxp3-eGFP+ cells into GITR-Lo and GITR-Hi bins are shown in Extended Data Figure 5B. Flow analysis of GITR expression in the sorted populations is shown in Extended Data Figure 5C.

☒ Tick this box to confirm that a figure exemplifying the gating strategy is provided in the Supplementary Information.
